# Mechanism and regulation of mitophagy in liver diseases: a review

**DOI:** 10.3389/fcell.2025.1614940

**Published:** 2025-06-27

**Authors:** Sen Liu, Libo Wang, Liuyang Zhu, Tianyu Zhao, Pinsheng Han, Fengying Yan, Xiaoliang Wang, Chunli Li, Ze Wang, Bao-feng Yang

**Affiliations:** ^1^ Department of Pharmacology, Shenyang Pharmaceutical University, Shenyang, China; ^2^ State Key Laboratory of Druggability Evaluation and Systematic Translational Medicine, Tianjin Institute of Pharmaceutical Research, Tianjin, China; ^3^ Department of Hepatobiliary Sugery, Tianjin First Central Hospital, Tianjin, China; ^4^ School of Medicine, Nankai University, Tianjin, China; ^5^ School of Pharmacy, Shenzhen Technology University, Shenzhen, China; ^6^ School of Pharmacy, Harbin Medical University, Harbin, China

**Keywords:** mitochondria, mitophagy, liver disease, DILI, NAFLD

## Abstract

Mitochondria are vital for the proper operation of healthy eukaryotic cells. Mitophagy, a specialized form of autophagy that targets damaged or surplus mitochondria, plays a key role in both the normal functioning and disease-related processes within the liver. This review aims to explore the main mechanisms underlying the initiation of mitophagy and its importance in various liver conditions, such as alcoholic liver disease, drug-induced liver injury, non-alcoholic fatty liver disease, viral hepatitis, and cancer. Gaining insight into these mechanisms can help overcome the obstacles related to harnessing mitophagy as a therapeutic strategy in clinical practice.

## 1 Introduction

Autophagy, more specifically macroautophagy, is marked by the formation of a double-membrane structure known as an autophagosome. These structures deliver their enclosed cargo to lysosomes, forming autolysosomes where lysosomal enzymes degrade the contents (Xie and Klionsky, 2007; Levine and Kroemer, 2008). Autophagic degradation can be divided into two main categories: nonselective and selective. Nonselective autophagy generally occurs in response to nutrient deprivation, resulting in the broad breakdown of cytoplasmic components to supply cells with necessary nutrients for survival. Conversely, selective autophagy targets specific substrates such as misfolded protein aggregates, dysfunctional or excess organelles, endoplasmic reticulum (ER), lipid droplets, and pathogens like bacteria and viruses.

Mitochondria are essential cellular organelles that play a role in maintaining ion and energy balance, overall cellular homeostasis, and programmed cell death. Their importance has led to the development of highly regulated mechanisms for ensuring mitochondrial quality control. Mitochondrial damage not only compromises their own function but can also negatively impact other cellular components, including organelles, proteins, and membranes. Selective autophagy targeting mitochondria, termed mitophagy, serves to eliminate damaged mitochondria, thus preserving cellular stability (Pickles et al., 2018). This review will delve into the growing relevance and regulatory pathways of mitophagy in liver disease contexts.

## 2 Mitophagy receptors

**FIGURE 1:**
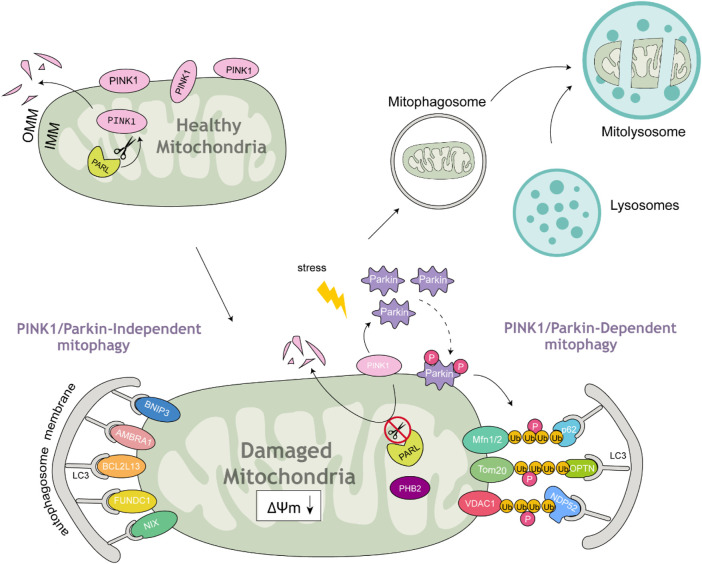
Parkin-dependent and Parkin-independent mitophagy. Under healthy mitochondrial conditions, PINK1 is anchored at the OMM and continuously imported from the cytosol to the IMM. It is then cleaved by mitochondrial proteases and translocated back to the cytosol. Once mitochondria are damaged under stress accompanied by MMP loss, the import of PINK1 is abolished; therefore, it accumulates on the OMM, leading to the recruitment and phosphorylation of Parkin. Subsequently, Parkin polyubiquitinates OMM proteins to form ubiquitin chains that bind to cargo receptors. These receptors interact with LC3 to form double-membrane mitophagosomes. Mitophagosomes fuse with lysosomes to form mitolysosomes, which then degrade damaged mitochondria.

Mitophagy is a specialized type of autophagy that selectively removes damaged mitochondria by linking mitochondrial components to the autophagic machinery (Pickles et al., 2018). Mitophagosomes usually have a double membrane, and the membrane of the isolated mitochondria remains intact. After fusion with mitolysosomes, the degradation of mitochondria and the corresponding ultrastructural degradation begin and complete within the mitolysosome. What are the signals or markers that direct substrates into the mitophagy pathway? To date, two categories of autophagy receptors have been identified: those reliant on ubiquitin signaling and those that operate independently of it. Ubiquitin-dependent mitophagy receptors (UDMRs) lack a transmembrane domain (TMD) but contain a ubiquitin-binding domain (UBD). Accumulating evidence indicates that ubiquitin plays a critical role in signaling for the autophagic degradation of protein aggregates and other cellular components (Kirkin et al., 2009). Among the most extensively studied UDMRs are sequestosome 1 (SQSTM1)/p62 (hereafter referred to as p62), optineurin (OPTN), nuclear domain 10 protein 52 kDa (NDP52), and prohibitin 2 (PHB2). These receptors usually feature one or two LIR (LC3-interacting region) motifs and a UBD located at the C-terminus. During mitophagy, selective cargoes are often tagged with ubiquitin. The UDMRs bind to their target molecules through the UBD and anchor themselves, along with these cargoes, to the autophagosome membrane via interactions between the LIR motif and LC3. In contrast, ubiquitin-independent mitophagy receptors (UIMRs) are pre-localized on organelle membranes and do not directly interact with ubiquitin. Examples of UIMRs include FUN14 domain containing 1 (FUNDC1), AMBRA1, Bcl2-like 13 (Bcl2L13), Bcl2/adenovirus E1B 19 kDa protein-interacting protein 3 (BNIP3), and Nix/BNIP3L. These proteins are associated with the mitochondrial membrane and directly interact with LC3 through their LIR motifs, recruiting the LC3-positive phagophore to the mitochondria to initiate mitophagy (Figure 1) (Table 1).

**TABLE 1:** Identified mitophagy receptors in mammalian cells.

Receptor	Interactor	Functions in mitophagy	Ref.
P62	Ubiquitin	P62/SQSTM1 contains on its C-terminus UBD, binding mostly to K63 and, to a lesser degree, to K48 polyubiquitin chains P62 directly interacts with LC3 through its LIR located in the interface between the N-terminus and C-terminal UBD. p62 recruits two subunits of a cullin-RING ubiquitin E3 ligase complex, Keap1 and Rbx1, to mitochondria. p62-Keap1-Rbx1 complex ubiquitinates mitochondria in parkin-independent mitophagy.	Geetha and Wooten (2002), Bjørkøy et al. (2006), Moscat et al. (2007), Kirkin et al. (2009), Yamada et al. (2018)
OPTN	Ubiquitin	OPTN contains UBD, the LIR motif	Belousov et al. (2021)
NDP52	Ubiquitin	NDP52 contains noncanonical LIR-motif, CC-domain (participates in auto-aggregation of NDP52) and C-terminal LIM-L domain binding mono- and polyubiquitin	Thurston et al. (2009), von Muhlinen et al. (2012), Xie et al. (2015), Furuya et al. (2018), Padman et al. (2019)
Prohibitin2	IMM	PHB2 may interact with LC3 through its LIR (YXXL) and recruit autophagic vacuoles to damaged mitochondria	Wei et al. (2017), Yan et al. (2020)
FUNDC1	OMM	The OMM-localized protein FUNDC1 regulates hypoxia-induced autophagy promoting mitophagy via interaction between its LIR and LC3	Liu et al. (2012)
AMBRA1	OMM/Parkin	Structurally, AMBRA1 is an intrinsically disordered protein. Similar to other LC3-interacting proteins, it contains the LRS (WXXL) on its C-terminus Parkin interacts with Ambra1 upon mitochondrial depolarization, and Ambra1 then further activates class III PI3K for autophagosome formation surrounding these mitochondrial clusters for their clearance	Van Humbeeck et al. (2011), Cianfanelli et al. (2015)
BCL2L13	OMM	Bcl2-L-13 is a single TMD OMM protein. Although BCL2L13 is described to comprise WXXL/I LRS-motifs, ULK1 was shown to be indispensable for BCL2L13 and LC3B association, implying that their interaction is indirect	Murakawa et al. (2015), Murakawa et al. (2019)
BNIP3	OMM	BNIP3 dimer phosphorylation at S17 is essential for LC3 binding via its LIR.	Hanna et al. (2012), Zhu et al. (2013), Shi et al. (2014)
NIX/BNIP3L	OMM/Ubiquitin	NIX comprises the LIR motif (WXXL) on its N-terminus to bind ATG8-like proteins, so that NIX can act as a receptor of mitophagy Parkin ubiquitinates NIX, which allows NIX to recruit NBR1, to the damaged mitochondria to help shuttle them to the mitophagosome for degradation	Novak et al. (2010), Gao et al. (2015)

## 3 Mitophagy signaling pathways

### 3.1 PINK1-parkin-dependent mitophagy

In 1998, Lemasters and colleagues observed that under nutrient-deprived conditions, a portion of mitochondria underwent spontaneous depolarization and were subsequently engulfed by lysosomes. This finding led to the introduction of the concept of mitochondrial autophagy (Lemasters et al., 1998). In 2005, they coined the term “mitophagy” to describe the selective autophagic degradation of mitochondria (Lemasters, 2005). Later, in 2008, Richard Youle’s team at NIH demonstrated that Parkin, an E3 ubiquitin ligase encoded by the Park2 gene, relocates to depolarized mitochondria, promoting their encapsulation by mitophagosomes and subsequent clearance via mitophagy (Narendra et al., 2008). Further research revealed that Parkin is recruited to damaged mitochondria through the action of phosphatase and tensin homolog-induced putative kinase 1 (PINK1), initiating the mitophagy process (Kawajiri et al., 2010; Matsuda et al., 2010; Narendra et al., 2010).

A key mechanism for the efficient removal of dysfunctional mitochondria involves a specialized form of autophagy called mitophagy. Among the signaling pathways involved in this process, the PINK1/Parkin pathway is the most well-characterized. In healthy mitochondria, PINK1, synthesized in the cytoplasm, is imported into mitochondria via molecular channels in the mitochondrial membrane and subsequently degraded by intramitochondrial proteases (Jin et al., 2010; Deas et al., 2011; Meissner et al., 2011; Greene et al., 2012). Mitochondrial damage disrupts the inner membrane potential, inhibiting PINK1 import and causing its accumulation on the outer mitochondrial membrane (OMM) (Matsuda et al., 2010; Narendra et al., 2010; Vives-Bauza et al., 2010). Accumulated PINK1 phosphorylates Ser65 residues on both ubiquitin and within Parkin’s UBD, enabling Parkin recruitment and attachment to mitochondria. PINK1-mediated Ser65 phosphorylation of ubiquitin also markedly enhances Parkin E3 ligase activity resulting in greater Parkin-induced OMM protein ubiquitination (Koyano et al., 2014). Parkin ubiquitinates a variety of OMM proteins including the mitochondrial fusion proteins mitofusin 1 (Mfn1) and Mfn2, the mitochondrial trafficking protein Miro1, the TOM20, and the voltage-dependent anion channel (VDAC). The degradation of Mfn1/Mfn2 induces mitochondrial fission, which may improve mitophagy efficiency as smaller mitochondria are more readily engulfed by mitophagosomes (Gegg et al., 2010; Geisler et al., 2010; Chan et al., 2011; Thomas et al., 2011). Additionally, the ubiquitination and degradation of Miro1 halt mitochondrial motility, isolating damaged mitochondria from healthy ones and facilitating their autophagic elimination (Wang et al., 2011).

### 3.2 P62/SQSTM1

In 1998, Shin initially discovered p62, highlighting its tendency to form aggregates. Based on this feature, he named it sequestosome 1 (SQSTM1) (Shin, 1998). The protein’s name originates from its molecular weight of 62 kDa. Human p62 comprises 440 amino acids and features several functional domains, including an N-terminal PB1 domain, a ZZ-type zinc finger domain, nuclear localization signals (NLS), a nuclear export signal (NES), LIR and KIR (KEAP1-interacting region) motifs, as well as a C-terminal UBD (Seibenhener et al., 2007; Komatsu and Ichimura, 2010; Pankiv et al., 2010). Among these domains, the LIR and UBD are most closely linked to autophagy processes. Later studies revealed that p62 acts as a selective autophagy substrate, undergoing continuous degradation through the autophagic pathway (Bjørkøy et al., 2005; Pankiv et al., 2007). Independent investigations by Outzen’s group and Komatsu’s group confirmed that p62 directly interacts with LC3 via the LIR motif, referred to as the LC3 recognition sequence (LRS) by Komatsu. This interaction is critical for the autophagic degradation of p62 (Pankiv et al., 2007; Ichimura et al., 2008). The LIR is a short acidic peptide sequence located between amino acids 334–344 in mouse p62, playing a key role in binding to LC3B. Further analysis showed that the LIR contains conserved aspartic acid and tryptophan residues essential for LC3B interaction. This motif facilitates interactions with all members of the mammalian ATG8 family, encompassing both the LC3 and GABARAP subfamilies.

Within its C-terminus, spanning amino acids 386–434, p62 noncovalently associates with mono- and poly-ubiquitinated proteins through its UBD. Due to its dual capacity to bind ubiquitinated proteins and LC3, p62 serves as a pivotal receptor protein for the selective autophagic clearance of ubiquitinated protein aggregates and organelles (Johansen and Lamark, 2011; Shaid et al., 2013). The C-terminal UBD of P62/SQSTM1 primarily interacts with K63-linked polyubiquitin chains and, to a lesser extent, K48-linked chains (Geetha and Wooten, 2002; Bjørkøy et al., 2006; Moscat et al., 2007; Kirkin et al., 2009).

### 3.3 Optineurin

Optineurin plays a vital role in preserving mitochondrial integrity. This protein comprises multiple functional domains, such as an NF-κB-essential molecule (NEMO)-like domain, a leucine zipper motif, a coiled-coil (CC) motif, a UBD, an LIR motif, and a zinc finger motif at the C-terminus. Using live-cell imaging, Wong et al. showed that optineurin is recruited to mitochondria that are depolarized or damaged by reactive oxygen species (ROS) in a Parkin-dependent manner (Wong and Holzbaur, 2014). Optineurin influences autophagosome formation by regulating the recruitment of the Atg12-5-16L1 complex (Bansal et al., 2018). Moreover, it acts as a selective autophagic receptor for substrate degradation by binding polyubiquitinated cargoes through its UBD (Ryan and Tumbarello, 2018). TBK1 phosphorylates optineurin at residues S177 and S473, strengthening its interaction with LC3 and reinforcing its association with phosphorylated ubiquitin (Wild et al., 2011; Ryan and Tumbarello, 2018).

### 3.4 NDP52/CALCOCO2

NDP52, alternatively referred to as CALCOCO2, includes a SKICH domain, which plays a crucial role in binding to TANK-binding kinase 1 (TBK1), LC3C, and mitochondrial poly(A) polymerase (MTPAP). Additionally, it possesses a noncanonical LIR, a coiled-coil (CC) domain involved in self-aggregation, and a zinc finger motif at the C-terminus that binds to both mono- and polyubiquitin chains (Thurston et al., 2009; von Muhlinen et al., 2012; Xie et al., 2015; Furuya et al., 2018; Padman et al., 2019). Although the zinc finger motif is responsible for polyubiquitin binding, the function of the SKICH domain is not yet fully understood. Furuya et al. demonstrated that NDP52 interacts with MTPAP via the SKICH domain. In the process of mitophagy, NDP52 penetrates depolarized mitochondria and associates with MTPAP in a manner dependent on the proteasome but independent of ubiquitin (Furuya et al., 2018).

### 3.5 Prohibitin2

The prohibitin (PHB) family is composed of two subunits PHB1 (32 KDa) and PHB2 (34 KDa), that physically associate with each other to form a large multimeric complex of approximately 1 MDa (Artal-Sanz and Tavernarakis, 2009; Hernando-Rodríguez and Artal-Sanz, 2018). Wei et al. demonstrated that the IMM protein PHB2 is an important mitophagy receptor involved in targeting mitochondria for autophagic degradation (Wei et al., 2017). PHB2 contains an LC3-interacting domain. In response to mitochondrial uncouplers and proteasome-mediated degradation of the OMM, binding of LC3 to PHB2 ensures mitochondrial clearance by recruiting the autophagic machinery of the IMM. Wei et al. showed that PHB2 is a mitochondrial receptor required for Parkin-mediated mitophagy in mammalian cells (Wei et al., 2017). Xiao et al. found that PHB2 plays a key role in cholestasis-mediated mitophagy *in vitro*. On the one hand, PHB2 binds to the autophagosome LC3 on damaged mitochondria via the LIR domain. On the other hand, PHB2 forms a ternary protein complex with p62 and LC3, leading to the loading of LC3 onto damaged mitochondria (Xiao et al., 2018). Yan et al. revealed that the PHB2-PARL-PGAM5-PINK1 axis is a novel pathway of PHB2-mediated mitophagy and that targeting PHB2 with the chemical compound FL3 is a promising strategy for cancer therapy (Yan et al., 2020).

### 3.6 FUNDC1

FUNDC1 has been recognized as a receptor that initiates mitophagy in a ubiquitin-independent manner under various stress conditions and during cell differentiation (Li G. et al., 2021). Like other mitophagy receptors, FUNDC1 possesses a canonical LIR motif in its N-terminal cytosolic region, which facilitates interaction with Atg8 family proteins and induces the selective removal of mitochondria (Wang and Wang, 2023). Initially identified in 2012 as a hypoxia-induced mitophagy receptor, FUNDC1 is phosphorylated by SRC kinase at Y18 under normal physiological conditions (Liu et al., 2012). During hypoxia, FUNDC1 undergoes dephosphorylation, strengthening its interaction with LC3 and promoting selective mitophagy (Liu et al., 2012). Chen et al. demonstrated that the mitochondrial phosphatase PGAM5 interacts with FUNDC1 and dephosphorylates it at Ser-13 in response to hypoxia or FCCP treatment, thereby enhancing mitophagy. Conversely, CK2 opposes this effect by phosphorylating FUNDC1 (Chen et al., 2014). Recovery under normoxia conditions was found to restore the mitochondrial membrane potential (MMP), which in turn led to the phosphorylation of FUNDC1 (Chen et al., 2014). Wu et al. presented a notable discovery that BCL2L1 suppresses FUNDC1-mediated mitophagy via its BH3 domain. In terms of mechanism, BCL2L1 binds to and suppresses PGAM5, a phosphatase located in the mitochondria, thereby preventing the dephosphorylation of FUNDC1 at Ser-13. As a result, this process halts mitophagy (Wu H. et al., 2014). Notably, ULK1 binds to FUNDC1 and phosphorylates it at Ser-17, increasing its affinity for LC3 and stimulating mitophagy (Wu W. et al., 2014). Thus, it appears that the phosphorylation or dephosphorylation of FUNDC1 at various sites can strengthen its interaction with LC3, thereby stimulating mitophagy. Additionally, Chen et al. discovered that the mitochondrial E3 ligase MARCH5, rather than Parkin, is involved in regulating hypoxia-induced mitophagy through the ubiquitylation and degradation of FUNDC1 (Chen et al., 2017).

### 3.7 AMBRA1

AMBRA1 (Activating molecule in Beclin1-regulated autophagy) is an intrinsically disordered protein (IDP) with a molecular weight of approximately 130 kDa. Its disordered nature enables it to function as a scaffold molecule, coordinating various intracellular processes with autophagy (Cianfanelli et al., 2015). Humbeeck et al. identified AMBRA1 as a Parkin-interacting protein that activates autophagy by stimulating the class III phosphatidylinositol 3-kinase (PI3K) complex, which is essential for phagophore formation. Notably, Parkin does not ubiquitinate AMBRA1 (Van Humbeeck et al., 2011). Nazio et al. reported that under non-autophagic conditions, mTOR suppresses AMBRA1 through phosphorylation. However, upon autophagy induction, AMBRA1 becomes dephosphorylated. In this scenario, AMBRA1 interacts with the E3 ligase TRAF6 to facilitate ULK1 ubiquitylation via LYS-63-linked chains, promoting its stabilization, self-association, and functionality (Nazio et al., 2013). Strappazzon et al. demonstrated that AMBRA1 binds to LC3 via its LIR motif, thereby regulating both Parkin-dependent and -independent mitochondrial clearance (Strappazzon et al., 2015). Rita et al. discovered HUWE1, an E3 ubiquitin ligase, as a crucial inducer in AMBRA1-dependent mitophagy, a process that occurs independently of the primary mitophagy receptors (Di Rita et al., 2018). Moreover, they demonstrated that the mitophagy function of AMBRA1 is post-translationally regulated and activated by HUWE1 through phosphorylation at serine 1,014. This modification, mediated by the IKKα kinase, triggers structural alterations in AMBRA1, enhancing its interaction with LC3/GABARAP (mATG8) proteins and thereby stimulating its role in mitophagy (Di Rita et al., 2018). Thus, Ambra1 might play a role in mitophagy through both Parkin-dependent and Parkin-independent pathways.

### 3.8 BCL2L13

Bcl2-L-13 (Bcl-2-like protein 13), a homolog of yeast Atg32, is a single transmembrane domain OMM protein that was first discovered in mammals by the Otsu research group in 2015. Bcl2-L-13 interacts with LC3 through the WXXI motif, promoting mitochondrial fragmentation and Parkin-independent mitophagy in HEK293 cells (Murakawa et al., 2015). In 2019, they found that BCL2L13 recruits the ULK1 complex during mitophagy, with the interaction between LC3B, ULK1, and BCL2L13 playing a critical role in this process (Murakawa et al., 2019). However, the *in vivo* function of Bcl2-L-13 remains unclear. More recent studies in 2024 demonstrated that BCL2L13-deficient mice and knockin mice with a mutated Ser272 (to Ala) exhibited left ventricular dysfunction under pressure overload due to defective mitochondrial fission and mitophagy. Attenuation of mitochondrial fission and mitophagy led to impairment of ATP production in these mouse hearts. Furthermore, Murakawa et al. found that AMPKa2 was identified as the kinase responsible for phosphorylating BCL2L13 at Ser272, underscoring its significance in maintaining cardiac function (Murakawa et al., 2024).

### 3.9 BNIP3 and NIX/BNIP3L

Bnip3 (Bcl2/adenovirus E1B 19 kDa protein-interacting protein 3) is an atypical BH3-only protein that is known to cause mitochondrial dysfunction and cell death (Hanna et al., 2012). The protein is embedded in the OMM through its C-terminal TMD, while its N-terminus is exposed to the cytosol. The C-terminal TMD plays a critical role in directing Bnip3 to the mitochondria, facilitating homodimer formation, and enabling its proapoptotic functions (Chen et al., 1997; Ray et al., 2000; Kubli et al., 2008). Notably, the N-terminus of Bnip3 includes a WXXL-like motif, which could play a key role in interacting with proteins from the Atg8 family. The interaction between Bnip3 and Atg8 proteins, such as LC3, may function to anchor mitochondria to autophagosomes, thus facilitating their degradation. While BH3-only proteins are known to link apoptotic and autophagic pathways, the mechanisms governing this cross-talk and its functional implications remain to be fully elucidated. Zhu et al. demonstrated that the phosphorylation of serine residues at positions 17 and 24, which are located near the Bnip3 LIR motif, enhances its interaction with specific Atg8 family members, namely, LC3B and GATE-16 (Zhu et al., 2013).

NIX, also known as BNIP3L, has been demonstrated to play a crucial role in regulating erythrocyte maturation via the process of mitophagy (Sandoval et al., 2008). Additionally, this research may offer a deeper understanding of the molecular mechanisms involved in mitochondrial quality control through mitophagy. Novak et al. demonstrated that the mitochondrial protein Nix acts as a selective autophagy receptor by interacting with LC3/GABARAP proteins, which are essential for the expansion of autophagosomal membranes (Novak et al., 2010). In cultured cells, Nix attracts GABARAP-L1 to impaired mitochondria via its amino-terminal LIR. Consequently, Nix serves as an autophagy receptor, facilitating mitochondrial clearance following mitochondrial damage and during erythrocyte differentiation. Rogov et al. provided evidence for a phosphorylation-dependent mechanism regulating the interaction between Nix and LC3B. Their findings, using isothermal titration calorimetry and NMR, revealed that phosphorylation of serines 34 and 35 in the Nix LIR increases its binding affinity to LC3B by approximately one hundredfold. This results in the formation of a significantly more stable and rigid complex compared to the non-phosphorylated form (Rogov et al., 2017). In patients with Parkinson’s disease resulting from the disruption of the PINK1/Parkin pathway, NIX functions as a neuroprotective factor by restoring mitophagy. Koentjoro et al. showed that in patients with PD caused by PINK1/Parkin pathway disruption, NIX acts as a neuroprotective agent, rescuing mitophagy and increasing neuronal cell and fibroblast viability (Koentjoro et al., 2017). Yuan et al. revealed the role of BNIP3L/NIX in mitophagy triggered by cerebral ischemia-reperfusion (I-R) injury. In mice, Bnip3l knockout led to defective mitophagy and worsened I-R-induced brain damage, an effect that could be reversed by overexpression of BNIP3L (Yuan et al., 2017).

## 4 Mitophagy in liver diseases

**FIGURE 2:**
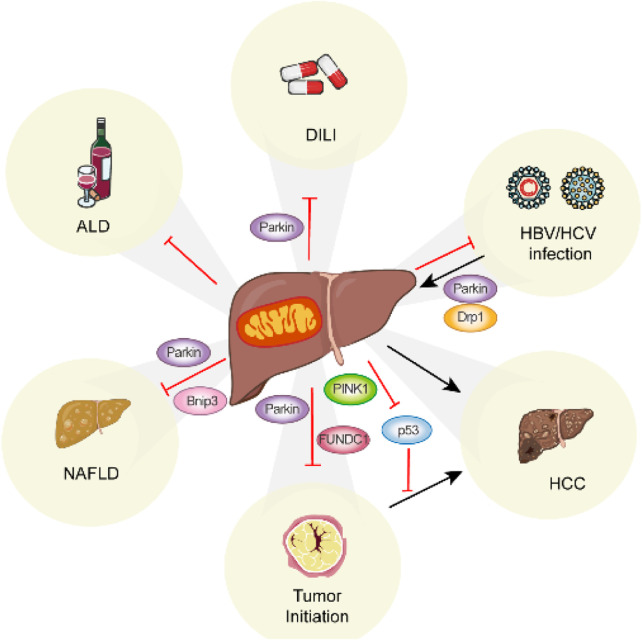
Summary of the role of mitophagy in liver disease. Mitophagy occurs in various liver diseases via distinct mitophagy regulators.

Research has demonstrated that multiple mechanisms of mitophagy are triggered under various stress conditions, as detailed in previous reviews (Choubey et al., 2021; Li S. et al., 2021). Here, we provide a summary of recent discoveries related to mitophagy-mediated pathways and their roles in the development of liver diseases (Figure 2) (Table 2).

**TABLE 2:** Mitophagy-related therapeutic targets in liver disease.

Disease	Regulators	Mitophagy pathway	Ref.
ALD	Pre-endurance training	Reduced BNIP3-mediated mitophagy	Ma et al. (2014)
	Ethanol	Activation of the PINK1-Parkin pathway	Eid et al. (2016)
	Fucoidan	Against ethanol-induced over-activated mitophagy. (PINK1/Parkin)	Zhao et al. (2021a)
DILI	Amitriptyline	Amitriptyline impaired mitophagy and increased susceptibility to APAP.	Baulies et al. (2015)
	PINK1 and Parkin double KO	APAP-induced mitophagy was significantly impaired in PINK1-Parkin DKO mice	Wang et al. (2019)
NAFLD	Quercetin	Quercetin alleviated hepatic steatosis by enhancing frataxin-mediated PINK1/Parkin-dependent mitophagy	Liu et al. (2018b)
	Mst1 knockdown	Mst1 knockdown reversed Parkin-related mitophagy and the latter protected mitochondria and hepatocytes against HFD challenge	Zhou et al. (2019b)
	Liraglutide	Liraglutide ameliorates NASH by inhibiting NLRP3 inflammasome and pyroptosis activation via PINK1/Parkin-dependent mitophagy	Yu et al. (2019)
	Exenatide	The expression of LC3A/B-II/I, Beclin-1, Parkin, and BNIP3L increased significantly after Exenatide treatment	Shao et al. (2018)
	Corilagin	Corilagin stimulates Parkin-mediated mitosis to relieve NAFLD.	Zhang et al. (2019)
	Cyanidin-3-O-glucoside	Cyanidin-3-O-glucoside stimulates Parkin-mediated mitosis to relieve NAFLD.	Li et al. (2020)
	Caloric restriction and metformin	Changes were enhanced in the CR + Met group for the mitophagy marker BNIP3	Linden et al. (2015)
	Sirtuin 3	Sirtuin 3 overexpression protected hepatocytes against mitochondrial apoptosis via promoting Bnip3-required mitophagy	Li et al. (2018)
	Akebia saponin D	Activation of BNip3 via ASD may offer a new strategy for treating NAFLD.	Gong et al. (2018)
HBV	HBV	HBV stimulated the gene expression of Parkin, PINK1, and LC3B and induced Parkin recruitment to the mitochondria	Kim et al. (2013a)
	HBx	HBx regulates diverse aspects of Parkin, enhancing mitophagy in starvation	Huang et al. (2018)
	HBx	HBx induced BNIP3L-dependent mitophagy	Chen et al. (2020)
	Thyroid Hormone	TH/PINK1/Parkin pathway has a critical role in protecting hepatocytes from HBx-induced carcinogenesis	Chi et al. (2017)
	MARCH5	Mitochondria ubiquitin ligase, MARCH5 resolves HBx protein aggregates in the liver pathogenesis	Yoo et al. (2019)
HCV	HCV	HCV infection stimulated Parkin and PINK1 gene expression	Kim et al. (2013b)
	HCV	HCV infection stimulated expression of Drp1 and its mitochondrial receptor, mitochondrial fission factor	Kim et al. (2014)
	Ginsenoside Rg3	G-Rg3 restores the HCV-induced Drp1–mediated aberrant mitochondrial fission process, thereby resulting in suppression of persistent HCV infection	Kim et al. (2017)
	NS5A	The expression of HCV NS5A in the hepatoma cells triggered hallmarks of mitophagy including mitochondrial fragmentation, loss of MMP, and Parkin translocation to the mitochondria	Jassey et al. (2019)
HCC	FUNDC1 knockout FUNDC1 overexpression	FUNDC1–mediated mitophagy suppresses HCC.	Li et al. (2019)
	Alantolactone	Alantolactone induces apoptosis through inhibition of PINK1-mediated mitophagy	Kang et al. (2019)
	PINK1	Mitophagy controls the activities of tumor suppressor p53 to regulate hepatic cancer stem cells	Liu et al. (2017a)
	MALAT1 knockdown	MALAT1 knockdown cells showed reduced expression of mitophagy markers, particularly PINK1, SQSTM1/p62, NDP52, BNIP3, and LC3B-I/II.	Zhao et al. (2021b)
	STOML2	STOML2 potentiates metastasis of HCC by promoting PINK1-mediated mitophagy	Zheng et al. (2021)
	Oroxylin A (CDK9 inhibitor)	Oroxylin A showed strong therapeutic potential against HCC by downregulating PINK1-PRKN-mediated mitophagy	Yao et al. (2022)
	YAP	Yap promotes HCC metastasis and mobilization by regulation of JNK/Bnip3/SERCA/CaMKII pathways	Shi et al. (2018)

### 4.1 Mitophagy in ALD

Alcohol-associated liver diseases (ALDs) pose a major global health challenge (Gao and Bataller, 2011; Weil et al., 2016; Axley et al., 2019; Aslam and Kwo, 2023; Danpanichkul et al., 2024). ALD often starts with asymptomatic steatosis but can silently progress to nonalcoholic steatohepatitis (NASH), fibrosis, and end-stage liver disease, including irreversible cirrhosis and HCC (Gao and Bataller, 2011). Chronic alcohol consumption leads to mitochondrial dysfunction in the liver and impairs the synthesis of mitochondrial respiratory complex proteins (Cunningham et al., 1990; Khambu et al., 2017; Lu et al., 2021). Ethanol is metabolized into acetaldehyde by alcohol dehydrogenase and CYP2E (Farré et al., 2009; Ding et al., 2010; Wu et al., 2012; Thomes et al., 2013), and the subsequent buildup of acetaldehyde has been shown to stimulate autophagy. Ethanol metabolism also enhances oxidative stress through mechanisms such as altering the NADH/NAD + ratio and causing mitochondrial damage (Lumeng and Crabb, 2000). Long-term ethanol exposure may induce mtDNA strand breaks and compromise its structural integrity (Cahill et al., 1999; Mansouri et al., 1999; Axley et al., 2019). As a compensatory mechanism against various types of mitochondrial damage, mitophagy is activated in ALD in response to mitochondrial depolarization and mtDNA damage. This process facilitates hepatic ethanol metabolism and protects cells from death by selectively eliminating damaged mitochondria, thereby reducing oxidative stress.

The impact of alcohol on mitophagy is determined by the duration and level of alcohol exposure. Mitophagy acts as a protective mechanism in ALD by removing dysfunctional mitochondria, a process that has been observed in both acute and chronic alcohol consumption (Ding et al., 2011; Williams et al., 2015a; Zhao H. et al., 2021). For example, Ma et al. found that pre-endurance training reduces the accumulation of damaged mitochondria caused by acute alcohol intake and alleviates BNIP3-mediated mitophagy, thereby restoring the balance between mitophagy and mitochondrial damage (Ma et al., 2014). Eid et al. demonstrated increased mitophagy in hepatocytes in an acute alcohol abuse rat model, evidenced by elevated LC3 puncta formation and colocalization of Parkin and LC3 with mitochondrial and lysosomal markers (Eid et al., 2016). These findings provide direct morphological evidence linking PINK1-Parkin pathway activation to an enhanced phagocytic response of hepatocytes against ethanol toxicity. Ethanol-induced hepatic mitophagy may serve as a prosurvival mechanism with potential therapeutic implications. Interestingly, impaired mitophagy is frequently reported in chronic alcohol consumption (Eid et al., 2013; Yu et al., 2016; Gao et al., 2019). Enhancing mitophagy function to mitigate ALD is a viable strategy (Tang et al., 2012; Yu et al., 2016; Gao et al., 2019; Lu et al., 2021). Alternatively, targeting excessive mitophagy could represent another therapeutic approach. Zhao et al. suggested that fucoidan pretreatment protects against ALD and over-activated mitophagy, maintaining mitochondrial homeostasis (Zhao H. et al., 2021). The differing mitophagy phenomena observed in acute *versus* chronic ALD models can be explained as follows: immediately after alcohol administration, extensive depolarization of hepatocyte mitochondria promotes hepatic ethanol metabolism, triggering regulatory signals for mitophagy as a compensatory response (Lemasters and Zhong, 2018). However, chronic alcohol intake results in a significant accumulation of dysfunctional mitochondria exceeding the processing capacity of mitophagy. Decompensated mitophagy leads to the release of mitochondrial damage-associated molecular patterns (mtDAMPs), promoting inflammation and fibrosis, further impairing liver mitophagy and advancing ALD progression (Lemasters and Zhong, 2018). These findings indicate that mitophagy plays a protective role in ALD and imply that targeting mitophagy could serve as a potential therapeutic strategy for ALD (Williams and Ding, 2015b; a;Chao and Ding, 2019).

It is important to note that the role of Drp1 and mitochondrial fission in regulating mitophagy and liver energy metabolism warrants further investigation in the context of ALD (Uchida et al., 1984; Han et al., 2017; Chao et al., 2018; Palma et al., 2019; Zhou H. et al., 2019).

### 4.2 Mitophagy in drug-induced liver injury

Drug-induced liver injury (DILI) is the leading cause of acute liver failure (ALF) in the United States and Europe. Additionally, it is a significant reason for drug discontinuation and high failure rates during drug development (Lee, 2013; Ye et al., 2018). A variety of medications can lead to DILI, including antitumor chemotherapy drugs, anti-tuberculosis agents, antipyretic analgesics, immunosuppressants, hypoglycemic therapies, and antibacterial, antifungal, and antiviral drugs. Acetaminophen (APAP), a widely used antipyretic and analgesic in the United States, is safe when administered at therapeutic doses but can induce liver damage and ALF if overdosed in humans and animals (Larson et al., 2005; Jollow, 2024). The metabolism of APAP is primarily catalyzed by cytochrome P450 enzymes (Potter et al., 1973), with N-acetyl-p-benzoquinone imine (NAPQI) being the key reactive metabolite associated with hepatotoxicity (Dahlin et al., 1984). The metabolite can reduce liver glutathione (GSH) levels and alter cellular proteins. While GSH binding occurs spontaneously, it may also be facilitated by GSH-S-transferases. The binding to proteins results in oxidative stress and mitochondrial dysfunction (McGill and Jaeschke, 2013). It is now well recognized that mitochondrial dysfunction plays a crucial role in the downstream signaling pathways following APAP overdose (Ramachandran and Jaeschke, 2018). While NAPQI can bind and detoxify GSH under normal conditions, excessive unbound NAPQI covalently binds to mitochondrial proteins, forming adducts that disrupt the electron transport chain (ETC.) and cause electron leakage and oxidative stress in hepatocytes (Kon et al., 2004; Cover et al., 2005; Ramachandran et al., 2011).

Mitochondria are central regulators of cell death and liver injury caused by various drugs (Jaeschke et al., 2012; Pessayre et al., 2012). The timely elimination of damaged mitochondria is essential for preventing DILI. Research has demonstrated that mitophagy is activated, as evidenced by increased Parkin translocation to mitochondria, ubiquitination of mitochondrial proteins, sequestration of impaired mitochondria into autophagosomes, and degradation of mitochondrial proteins in primary mouse liver hepatocytes (Williams et al., 2015b; Wang et al., 2019). Pharmacological induction of autophagy using rapamycin nearly eliminated APAP-induced liver injury in mice, whereas inhibition of autophagy with 3-methyladenine or chloroquine exacerbated APAP-induced hepatotoxicity (Ni et al., 2012; Shan et al., 2019). Baulies et al. reported that mice with lysosomal dysfunction exhibited higher mortality rates following APAP overdose due to defective fusion of mitochondria-containing autophagosomes with lysosomes (Baulies et al., 2015). These findings suggest that autophagy/mitophagy plays a protective role in mitigating excessive APAP-induced hepatotoxicity. Interestingly, compared to the pronounced mitophagy disruption observed in PINK1/Parkin double knockout (DKO) mice after APAP administration, mice with either PINK1 or Parkin single knockouts displayed mild mitochondrial defects. However, acute knockout of Parkin accelerated APAP-induced liver injury in mice, suggesting that mice did not have enough time to adapt to the acute loss of Parkin under acute knockout of Parkin time window (Shan et al., 2019). Moreover, Parkin knockout mice showed resistance to APAP-induced liver injury (Wang et al., 2019). These data indicate that PINK1 and Parkin may compensate for each other to maintain mitophagy and protect against DILI. Although PINK1/Parkin-mediated mitophagy acts as a protective mechanism against APAP-induced hepatotoxicity, additional Parkin-independent pathways merit further investigation.

### 4.3 Mitophagy in NAFLD

Nonalcoholic fatty liver disease (NAFLD) is the hepatic manifestation of metabolic syndrome, encompassing a range of liver conditions from simple steatosis to NASH, fibrosis, cirrhosis, and HCC in advanced stages (Younes and Bugianesi, 2019). Pathologically, it is marked by hepatocyte ballooning, inflammatory infiltration, collagen deposition, and hepatocyte death (Liu J. et al., 2018). NAFLD develops due to increased lipogenesis caused by abnormal lipid metabolism following excessive free fatty acid uptake by hepatocytes (Lee et al., 2019). Given the essential role of mitochondria in fatty acid metabolism and energy production, mitochondrial dysfunction is considered a key feature of NAFLD (García-Ruiz et al., 2013; Simões et al., 2018).

A growing body of evidence associates impaired mitophagy with NAFLD. Studies using high-fat diet (HFD)-induced mouse models and cultured cells treated with oleic acid (OA) or palmitic acid (PA) have demonstrated that restoring defective mitophagy in hepatocytes improves metabolic outcomes. Diet-induced NAFLD leads to autophagic arrest in hepatocytes, contributing to oxidative stress, mitochondrial dysfunction, and insulin resistance. Conversely, targeted deletion of ALCAT1 in mice prevents NAFLD onset, restores mitophagy, enhances mitochondrial structure, preserves mtDNA integrity, and boosts oxidative phosphorylation (Wang et al., 2015). Pharmacological enhancement of PINK1/Parkin-dependent mitophagy through quercetin, a plant flavonol, alleviates HFD-induced liver disease in a 10-week feeding mouse model (Liu P. et al., 2018). Additionally, Zhou et al. found that macrophage stimulating 1 (Mst1), identified as a novel upstream regulator of mitophagy, exacerbates apoptosis in cardiac and cancer tissues by suppressing mitophagy activity (Zhou T. et al., 2019). Knockdown of Mst1 reverses Parkin-related mitophagy, protecting mitochondria and hepatocytes against HFD challenges. Liraglutide, a long-acting GLP-1 analog, reduces lipid accumulation, inhibits the activation of the nucleotide-binding oligomerization domain-like receptor pyrin domain-containing protein 3 (NLRP3) inflammasome and pyroptosis, mitigates mitochondrial dysfunction and ROS generation, and promotes mitophagy in hepatocytes (Yu et al., 2019). Inhibition of mitophagy using 3-methyladenine or PINK1-targeted siRNA diminishes liraglutide-mediated suppression of inflammatory injury. Exenatide could alleviate oxidative stress damage and suppress the NLRP3 inflammasome by promoting the mitophagy pathway in the liver, thereby providing a protective role for the liver in NAFLD and diabetes within C57BL/6 mice (Shao et al., 2018). Compounds such as corilagin and cyanidin-3-O-glucoside stimulate Parkin-mediated mitophagy, mitigating NAFLD by inhibiting hepatic oxidative stress, NLRP3 inflammasomes, steatosis, and improving systemic glucose metabolism (Zhang et al., 2019; Li et al., 2020). Nevertheless, research on Parkin gene-deleted mice has yielded highly unexpected outcomes. Parkin gene-deleted mice exhibited resistance to weight gain, steatohepatitis, and insulin resistance (Kim et al., 2011). Partially, Parkin exerted this effect through the ubiquitin-mediated stabilization of the lipid transporter CD36 (Kim and Sack, 2012). Costa et al. discovered that intestinal lipid absorption is compromised in Park2 knockout mice, as indicated by elevated fecal lipids and decreased plasma triglycerides following an intragastric fat challenge (Costa et al., 2016). This dialectically demonstrates that Parkin plays an important role in regulating lipid absorption in addition to mediating mitochondrial autophagy.

In addition, Bnip3-mediated mitophagy also plays a crucial role in regulating liver lipid metabolism and may protect against NAFLD progression. Loss of BNip3 increases hepatic lipid synthesis, associated with elevated ATP levels, reduced AMPK activity, and increased expression of lipogenic enzymes (Glick et al., 2012). Furthermore, the impairment of BNIP3-induced mitophagy also significantly contributes to the regulation of hepatic lipid metabolism, thereby facilitating the progression of NAFLD (Linden et al., 2015). Overexpression of Sirtuin3 protects hepatocytes from mitochondrial apoptosis by promoting BNip3-dependent mitophagy, reversing BNip3 expression and mitophagy activity via the ERK-CREB signaling pathway (Li et al., 2018). Akebia saponin D alleviates hepatic steatosis by targeting BNip3-mediated mitophagy, offering a potential therapeutic strategy for NAFLD (Gong et al., 2018).

### 4.4 Mitophagy in viral hepatitis

In recent years, viral hepatitis has become a major global health concern, affecting hundreds of millions of people and causing significant morbidity and mortality. Both Hepatitis B virus (HBV) and Hepatitis C virus (HCV) are strongly associated with chronic health complications. The majority of deaths attributed to viral hepatitis are caused by HBV and HCV, despite the overall morbidity linked to these infections (Lanini et al., 2019). Emerging research highlights an increasing connection between mitochondrial dysfunction and HBV/HCV infections (Simula and De Re, 2010; Fisicaro et al., 2017; Montali et al., 2023).

HBV infection affects approximately 350 million individuals worldwide, with chronic HBV being closely linked to HCC(Neuveut et al., 2010; Guerrieri et al., 2013; Chen and Tian, 2019; Torresi et al., 2019; Qian et al., 2024). The HBV genome consists of relaxed-circular DNA (rcDNA) approximately 3.2 kb in length, featuring a complete minus strand and an incomplete plus strand. This genome encodes four overlapping open reading frames (ORFs): C, P, S, and X. These ORFs produce functional viral proteins, including HBc and its derivatives such as the E antigen (HBe) and the 22-kDa precore protein (p22cr) from ORF C; Pol from ORF P; three types of surface antigens—L-HBs, M-HBs, and S-HBs—from ORF S; and the HBV X protein (HBx) from ORF X (Tsukuda and Watashi, 2020; Asandem et al., 2024). HBx plays a critical role in both hepatocarcinogenesis and HBV replication (Andrisani, 2013). The interaction between HBx and the mitochondrial protein HVDAC3 (human voltage-dependent anion channel 3) has been confirmed using standard *in vitro* and *in vivo* techniques (Rahmani et al., 2000). It is widely acknowledged that HBV exploits autophagy to enhance its replication efficiency (Sir et al., 2010; Khan et al., 2018). Kim et al. reported that HBV modifies mitochondrial dynamics by promoting fission and mitophagy, thereby reducing virus-induced apoptosis. Additionally, HBV stimulates the expression of Parkin, PINK1, and LC3B genes and induces the recruitment of Parkin to mitochondria (Kim et al., 2013a). Huang et al. demonstrated that HBx enhances PINK1/Parkin-mediated mitophagy under starvation conditions. HBx not only upregulates the expression of PINK1/Parkin genes but also accelerates Parkin recruitment to specific mitochondria (Huang et al., 2018). Chen et al. proposed a positive feedback mechanism wherein HBx induces BNIP3L-dependent mitophagy, which upregulates glycolytic metabolism and increases the cancer stemness of HCC cells both *in vivo* and *in vitro* (Chen et al., 2020). Chi et al. confirmed the protective effects of thyroid hormone (TH) against HBx-induced hepatocarcinogenesis through activation of the PINK1/Parkin pathway in hepatocytes (Chi et al., 2017). MARCH5, an E3 ligase located in the OMM, correlates positively with the survival rates of HCC patients (Yoo et al., 2019). HBx-induced ROS production, mitophagy were suppressed in the presence of high MARCH5 expression.

HCV is a significant human pathogen capable of inducing severe liver diseases, including acute and chronic hepatitis, cirrhosis, and HCC (Chu and Ou, 2021). This virus is a small, enveloped RNA virus with a single-stranded RNA genome encoding both structural and nonstructural proteins. The structural proteins, which form the viral particle, include the core protein and the envelope glycoproteins E1 and E2. In contrast, the nonstructural proteins include the p7 ion channel, the NS2-3 protease, the NS3 serine protease and RNA helicase, the NS4A polypeptide, the NS4B and NS5A proteins, and the NS5B RNA-dependent RNA polymerase (RdRp) (Moradpour et al., 2007; Fatima et al., 2014; Houghton, 2019).

HCV infection has been shown to influence mitophagy. Kim et al. reported that HCV-infected cells exhibit a higher number of mitophagosomes compared to uninfected cells. The presence of HCV-induced mitophagy was confirmed by the colocalization of LC3 puncta with Parkin-associated mitochondria and lysosomes (Kim et al., 2013b). Furthermore, Kim et al. demonstrated that HCV disrupts mitochondrial dynamics by promoting mitochondrial fission, followed by mitophagy, which helps mitigate HCV-induced apoptosis (Kim et al., 2014). Surprisingly, however, Hara et al. found that HCV core protein inhibits the transport of Parkin to affected mitochondria by interacting with Parkin and subsequently inhibiting mitophagy (Hara et al., 2014). The contradictory findings between the two studies may be due to experimental conditions, such as the presence or absence of CCCP (carbonyl cyanide m-chlorophenylhydrazone) in the culture and different post-infection times. Additionally, Kim et al. identified G-Rg3 as an inhibitor of HCV propagation. Its antiviral mechanism involves restoring HCV-induced dysregulation of mitochondrial fission mediated by DRP1, leading to the suppression of chronic HCV infection (Kim et al., 2017). Jassey et al. highlighted the role of the NS5A in regulating cellular mitophagy. Specifically, the expression of NS5A in hepatoma cells induced key features of mitophagy, such as mitochondrial fragmentation, loss of MMP, and translocation of Parkin to the mitochondria (Jassey et al., 2019).

### 4.5 Mitophagy in liver cancer

HCC is the sixth most common malignancy and fourth leading cause of cancer-related death worldwide (Brown et al., 2023). The epidemiology of HCC is rapidly changing, with NAFLD increasingly contributing to fibrosis, cirrhosis, and HCC development (Ganesan and Kulik, 2023). Under these pathological conditions, mitochondria play a pivotal role in regulating energy production and determining cell survival or death. Damaged mitochondria produce excessive ROS, which impair DNA, proteins, and lipids, all contributing to the initiation and progression of HCC (Severi et al., 2010; Nakahira et al., 2011; Vera-Ramirez et al., 2011; Nickel et al., 2014; Schieber and Chandel, 2014; Sia et al., 2017).

HCC is a well-known inflammation-associated cancer, with over 90% of cases arising in the context of hepatic injury and inflammation. When mitochondria are damaged, ROS and mtDNA are released into the cytosol, activating innate immune responses that contribute to liver cancer initiation and progression (Nakahira et al., 2011; Bishayee, 2014). Li et al. observed that FUNDC1, a previously characterized mitophagy receptor, accumulates in most HCCs. They further demonstrated that specific knockout of FUNDC1 in hepatocytes promotes diethylnitrosamine (DEN)-induced HCC initiation and progression, whereas FUNDC1-overexpressing hepatocytes protect against HCC development (Li et al., 2019). Specifically, depletion of FUNDC1 in hepatocytes increases cytosolic mtDNA release and caspase-1 activation, leading to elevated secretion of proinflammatory cytokines such as interleukin-1β (IL1β) and hyperproliferation of hepatocytes. These findings highlight the protective role of FUNDC1-mediated mitophagy in suppressing HCC progression through regulation of mitochondrial quality control and inflammatory responses.

Interestingly, accumulating evidence suggests that mitophagy may paradoxically promote cancer growth and metastasis (Zong et al., 2016; Wang et al., 2021). Sesamol, a phenolic antioxidant compound abundant in sesame seeds, has been shown to inhibit mitophagy and autophagy by disrupting the PI3K Class III/Beclin-1 pathway (Liu Z. et al., 2017). Alantolactone, another compound, demonstrates therapeutic potential in liver cancer by suppressing PINK1/Parkin-mediated mitophagy (Kang et al., 2019). Liver cancer stem cells (LCSCs), a small subset of HCC cells responsible for self-renewal, differentiation, metastasis, and recurrence, exhibit activation of BNIP3L-mediated mitophagy. This process induces a metabolic shift toward glycolysis in LCSCs and HBx-expressing HCC cells, increasing the proportion of the LCSC population to maintain cancer stemness (Chen et al., 2020). Liu et al. revealed that mitophagy is essential for sustaining LCSCs by degrading mitochondria-associated p53. Otherwise, p53 would be activated by PINK1 to suppress the expression of NANOG, a key factor in maintaining stemness (Liu K. et al., 2017). Mitophagy also plays a critical role in the progression of benign tumors to malignant ones. Inhibition of mitophagy increases the level of PINK1-mediated phosphorylated TP53, which translocates to the nucleus to suppress NANOG expression, thereby preventing the transition from benign to malignant liver tumors (Qian et al., 2018). The long noncoding RNA (lncRNA) MALAT1 (metastasis-associated lung adenocarcinoma transcript 1), typically enriched in the nucleus, was found to be aberrantly localized in the mitochondria of hepatoma cells. Zhao et al. demonstrated that MALAT1-deficient cells exhibit reduced expression of mitophagy markers such as PINK1, p62, NDP52, BNIP3, and the LC3B-II/I ratio (Zhao Y. et al., 2021). Compared with paired adjacent normal tissues, IMM protein STOML2 expression was increased in HCC. Zheng et al. found that STOML2 enhances mitophagy by stabilizing PINK1, promoting HCC metastasis and modulating the response to lenvatinib (Zheng et al., 2021). CDK9 (cyclin-dependent kinase 9), a catalytic subunit of the transcription elongation factor P-TEFb, is considered a promising target for cancer therapy. Yao et al. reported that inhibition of CDK9 disrupts the SIRT1-FOXO3-BNIP3 axis and the PINK1-PRKN pathway, effectively blocking mitophagy initiation in HCC (Yao et al., 2022). Yap, a transcriptional coactivator, is significantly upregulated in HCC and promotes cell migration. Shi et al. discovered that Yap inhibits BNIP3-mediated excessive mitophagy, improving mitochondrial function and ATP storage, thereby facilitating HCC progression (Shi et al., 2018).

Overall, similar to general autophagy, it seems that depending on the stage of tumorigenesis, mitophagy also plays a dual role in HCC development. Mitophagy prevents HCC initiation by suppressing the accumulation of dysfunctional mitochondria, cellular oxidative stress, genomic instability, and inflammation. Once carcinogenesis is initiated, mitophagy is highly activated to support tumor cell metabolic demands and promote HCC progression. Therefore, targeted therapy that selectively inhibits mitophagy in actively proliferating tumor cells while enhancing mitophagy in adjacent normal cells may be a promising but challenging therapeutic direction for HCC in the future.

## 5 Analysis of mitophagy

Mitophagy is a dynamic process that begins with phagocytosis by mitophagosomes and ends with mitolysosomal degradation. It is important to use multiple analytical methods when monitoring mitophagy *in vitro* and *in vivo* models. The most widely used methods currently used to study and quantify mitophagy include electron microscopy (EM), fluorescence microscopy of co-localization of mitochondria with mitophagosomes or mitolysosomes, mitochondrial mass determination, and a range of newly developed pH-sensitive fluorescent probes (Williams and Ding, 2018).

EM is one of the best tools to study mitophagy because it provides visualization of double-membrane mitophagosomes. However, due to the limited number of cells and sections, it is difficult to quantify mitophagy activation by EM. Colocalization of mitochondria with mitophagosomes and mitolysosomes by fluorescent co-labeling provides quantitative results in a large number of cells. However, quantifying the co-localization of mitochondria with autophagosomes or lysosomes is subjective and thus requires the use of consistent criteria across experiments. The final step of the mitophagic degradation process can be monitored by measuring mitochondrial mass using MitoTracker staining, flow cytometry, and Western blotting using antibodies against mitochondrial proteins. However, MitoTracker staining depends on MMP and may not stain damaged mitochondria. Mitochondrial proteins, especially OMM proteins, are degraded by autophagy and proteasomes (Tanaka et al., 2010; Chan et al., 2011; Yoshii et al., 2011). Therefore, if mitochondrial quality is monitored by Western blotting, several mitochondrial proteins should be used, including inner membrane and matrix proteins. The development of pH-dependent fluorescent molecular tools has greatly improved the quality of traditional fluorescence imaging, making image-based methods more accurate and reproducible in monitoring mitophagy. Mito-QC is a pH-sensitive mitochondrial fluorescent probe consisting of a tandem mCherry-GFP tag fused to the mitochondrial targeting sequence of the OMM protein FIS1. Mito-QC mice allow monitoring of mitophagy *in vivo*. Under steady-state conditions, mito-QC displays both red and green fluorescence. When mitophagy is induced, mito-QC displays an mCherry red signal that is stabilized when mitophagy is delivered to lysosomes. The green GFP signal is quenched when mitophagy is delivered to lysosomes (McWilliams et al., 2016). However, since FIS1 is located on the OMM, it was unclear whether some mito-QC could be degraded by the ubiquitin proteasome system rather than mitophagy. To circumvent this possibility, Chan’s group developed a similar probe (Cox8-EGFP-mCherry) targeting the IMM protein Cox8 (Rojansky et al., 2016).

## 6 Mitophagy interacts with other cellular stress responses

Accumulating evidence suggests that mitophagy plays a regulatory role in other cellular stress responses. As a form of selective autophagy, mitophagy can induce autophagic cell death when mitochondrial clearance is excessive. Certain drugs or compounds have been shown to target mitochondria, leading to excessive mitophagy and autophagic cell death (Meyer et al., 2018; Yao et al., 2018; Huang et al., 2021). Mitochondria and ER are two important organelles in cells that are closely linked in function and structure. It was found that about 20% of the mitochondrial surface is in contact with the ER membrane, called mitochondria-associated ER. Vesicle-associated membrane-associated protein (VAPB) is a recombinant protein on the ER membrane; it binds to the OMM protein tyrosine phosphatase protein 51 (PTPIP51) to form a tether that connects the ER to the mitochondria and keeps them at a distance of 10–30 nm (Gomez-Suaga et al., 2017). When VAPB or PTPIP51 is overexpressed, the contact surface between mitochondria and the ER increases, leading to mitochondrial calcium overload, MPTP opening, release of cytochrome c and ROS, and induction of cell apoptosis. When the expression of VAPB and PTPIP51 is reduced, the distance between mitochondria and the ER becomes longer, resulting in insufficient Ca2+ in mitochondria, inhibition of the TCA cycle, and reduced ATP production. For more details, see the review by Liu et al. (Liu et al., 2022).

Accumulating evidence suggests that mitophagy is a negative regulator of NLRP3 inflammasome-mediated pyroptosis. In NASH, liraglutide can induce PINK1-Parkin-mediated mitosis, thereby limiting ROS production and inhibiting NLRP3-mediated hepatocyte pyroptosis (Yu et al., 2019). Wu et al. showed that betulinic acid can restore autophagic flux after injury, thereby inducing mitophagy to eliminate ROS accumulation and inhibit NLRP3-mediated pyroptosis (Wu et al., 2021).

## 7 Summary and future perspectives

In conclusion, mitophagy acts as a key regulatory mechanism for eliminating damaged mitochondria, allowing cells to preserve their stability and functionality. This process provides protective effects in the development and progression of various liver diseases, such as ALD, DILI, NAFLD, and viral hepatitis. On the other hand, mitophagy demonstrates a dual nature in liver tumorigenesis and cancer progression, functioning as both a protective and potentially harmful factor depending on the specific context. Although the PINK1/Parkin-dependent pathway is one of the most extensively studied mechanisms of mitophagy, the role of PINK1/Parkin-independent pathways in liver pathogenesis remains to be fully elucidated. Recent progress has enhanced our understanding of how mitophagy manages mitochondrial damage in liver diseases. Nevertheless, the interactions between different mitophagy-mediated pathways and the exact mechanisms by which mitophagy influences disease initiation and progression are still unclear and require further exploration. Numerous studies have demonstrated the therapeutic potential of strategies targeting mitophagy, and related drugs have also shown good prospects in the treatment of various diseases (Table 3). Future studies in this field are expected to expand our knowledge, facilitating the development of more effective strategies for new drug discovery and clinical treatments. These endeavors offer promising potential for advancing therapeutic interventions targeting mitophagy in liver diseases.

**TABLE 3:** Pharmacological targeting of mitophagy.

Molecule name	Functional mechanism	Species	Outcome	Clinical trial	Ref.
Urolithin A	Promote mitophagy and activate Nrf2/ARE signaling	Mouse	Reduced oxidative stress	Phase 2/Completed	Gao et al. (2022)
Metformin	Activate AMPK and restore the expression of UQCRC2	Mouse	Reduced ethanol-induced liver injury	Phase 4/Completed	Lu et al. (2021)
Deferiprone (DFP)	Induce mitophagy by iron chelation	Mouse	Decreased hepatic inflammation	Phase 4/Completed	Tong et al. (2023)
Icaritin	Recover MMP and inhibit mTOR complex I	Mouse	Prolonged survival time of mice at the advanced stage of HCC	Phase 2/Completed	Yu et al. (2020)
AT101	Inducing BNIP3 and NIX-mediated mitophagy	None	Inducing autophagic cell death	Phase 2	Meyer et al. (2018)
Ketoconazole	Inducing PINK1-Parkinmediated mitophagy	PDX model	Inducing apoptosis	Not Found	Chen et al. (2019)
Salidroside	Inducing Parkin-dependent mitophagy	Rats	Inhibiting apoptosis	Not Found	Zhang et al. (2018)
Liraglutide	Inducing PINK1-Parkinmediated mitophagy	None	Inhibiting pyroptosis	Phase 4	Yu et al. (2019)
TMP	Inducing PINK1-Parkinmediated mitophagy	Mouse	Inhibiting necroptosis	Not Found	Zhou et al. (2022)
Silibinin	Inducing PINK1-Parkinmediated mitophagy	None	Inhibiting ferroptosis	Not Found	Du et al. (2023)

## References

[B1] Andrisani O. M. (2013). Deregulation of epigenetic mechanisms by the hepatitis B virus X protein in hepatocarcinogenesis. Viruses 5, 858–872. 10.3390/v5030858 23507839 PMC3705300

[B2] Artal-Sanz M. Tavernarakis N. (2009). Prohibitin and mitochondrial biology. Trends Endocrinol. Metab. 20, 394–401. 10.1016/j.tem.2009.04.004 19733482

[B3] Asandem D. A. Segbefia S. P. Kusi K. A. Bonney J. H. K. (2024). Hepatitis B virus infection: a mini review. Viruses 16, 724. 10.3390/v16050724 38793606 PMC11125943

[B4] Aslam A. Kwo P. Y. (2023). Epidemiology and disease burden of alcohol associated liver disease. J. Clin. Exp. Hepatol. 13, 88–102. 10.1016/j.jceh.2022.09.001 36647400 PMC9840073

[B5] Axley P. D. Richardson C. T. Singal A. K. (2019). Epidemiology of alcohol consumption and societal burden of alcoholism and alcoholic liver disease. Clin. Liver Dis. 23, 39–50. 10.1016/j.cld.2018.09.011 30454831

[B6] Bansal M. Moharir S. C. Sailasree S. P. Sirohi K. Sudhakar C. Sarathi D. P. (2018). Optineurin promotes autophagosome formation by recruiting the autophagy-related Atg12-5-16L1 complex to phagophores containing the Wipi2 protein. J. Biol. Chem. 293, 132–147. 10.1074/jbc.M117.801944 29133525 PMC5766911

[B7] Baulies A. Ribas V. Núñez S. Torres S. Alarcón-Vila C. Martínez L. (2015). Lysosomal cholesterol accumulation sensitizes to acetaminophen hepatotoxicity by impairing mitophagy. Sci. Rep. 5, 18017. 10.1038/srep18017 26657973 PMC4676017

[B8] Belousov D. M. Mikhaylenko E. V. Somasundaram S. G. Kirkland C. E. Aliev G. (2021). The dawn of mitophagy: what do we know by now? Curr. Neuropharmacol. 19, 170–192. 10.2174/1570159X18666200522202319 32442087 PMC8033973

[B9] Bishayee A. (2014). The role of inflammation and liver cancer. Adv. Exp. Med. Biol. 816, 401–435. 10.1007/978-3-0348-0837-8_16 24818732

[B10] Bjørkøy G. Lamark T. Brech A. Outzen H. Perander M. Overvatn A. (2005). p62/SQSTM1 forms protein aggregates degraded by autophagy and has a protective effect on huntingtin-induced cell death. J. Cell Biol. 171, 603–614. 10.1083/jcb.200507002 16286508 PMC2171557

[B11] Bjørkøy G. Lamark T. Johansen T. (2006). p62/SQSTM1: a missing link between protein aggregates and the autophagy machinery. Autophagy 2, 138–139. 10.4161/auto.2.2.2405 16874037

[B12] Brown Z. J. Tsilimigras D. I. Ruff S. M. Mohseni A. Kamel I. R. Cloyd J. M. (2023). Management of hepatocellular carcinoma: a review. JAMA Surg. 158, 410–420. 10.1001/jamasurg.2022.7989 36790767

[B13] Cahill A. Stabley G. J. Wang X. Hoek J. B. (1999). Chronic ethanol consumption causes alterations in the structural integrity of mitochondrial DNA in aged rats. Hepatology 30, 881–888. 10.1002/hep.510300434 10498638 PMC2647744

[B14] Chan N. C. Salazar A. M. Pham A. H. Sweredoski M. J. Kolawa N. J. Graham R. L. (2011). Broad activation of the ubiquitin-proteasome system by parkin is critical for mitophagy. Hum. Mol. Genet. 20, 1726–1737. 10.1093/hmg/ddr048 21296869 PMC3071670

[B15] Chao X. Ding W. X. (2019). Role and mechanisms of autophagy in alcohol-induced liver injury. Adv. Pharmacol. 85, 109–131. 10.1016/bs.apha.2019.01.008 31307584 PMC7141786

[B16] Chao X. Wang S. Zhao K. Li Y. Williams J. A. Li T. (2018). Impaired TFEB-mediated lysosome biogenesis and autophagy promote chronic ethanol-induced liver injury and steatosis in mice. Gastroenterology 155, 865–879.e12. 10.1053/j.gastro.2018.05.027 29782848 PMC6120772

[B17] Chen G. Han Z. Feng D. Chen Y. Chen L. Wu H. (2014). A regulatory signaling loop comprising the PGAM5 phosphatase and CK2 controls receptor-mediated mitophagy. Mol. Cell 54, 362–377. 10.1016/j.molcel.2014.02.034 24746696

[B18] Chen G. Ray R. Dubik D. Shi L. Cizeau J. Bleackley R. C. (1997). The E1B 19K/Bcl-2-binding protein Nip3 is a dimeric mitochondrial protein that activates apoptosis. J. Exp. Med. 186, 1975–1983. 10.1084/jem.186.12.1975 9396766 PMC2199165

[B19] Chen Y. Chen H. N. Wang K. Zhang L. Huang Z. Liu J. (2019). Ketoconazole exacerbates mitophagy to induce apoptosis by downregulating cyclooxygenase-2 in hepatocellular carcinoma. J. Hepatol. 70, 66–77. 10.1016/j.jhep.2018.09.022 30287340

[B20] Chen Y. Tian Z. (2019). HBV-induced immune imbalance in the development of HCC. Front. Immunol. 10, 2048. 10.3389/fimmu.2019.02048 31507621 PMC6718466

[B21] Chen Y. Y. Wang W. H. Che L. Lan Y. Zhang L. Y. Zhan D. L. (2020). BNIP3L-Dependent mitophagy promotes HBx-Induced cancer stemness of hepatocellular carcinoma cells *via* glycolysis metabolism reprogramming. Cancers (Basel) 12, 655. 10.3390/cancers12030655 32168902 PMC7139741

[B22] Chen Z. Liu L. Cheng Q. Li Y. Wu H. Zhang W. (2017). Mitochondrial E3 ligase MARCH5 regulates FUNDC1 to fine-tune hypoxic mitophagy. EMBO Rep. 18, 495–509. 10.15252/embr.201643309 28104734 PMC5331199

[B23] Chi H. C. Chen S. L. Lin S. L. Tsai C. Y. Chuang W. Y. Lin Y. H. (2017). Thyroid hormone protects hepatocytes from HBx-induced carcinogenesis by enhancing mitochondrial turnover. Oncogene 36, 5274–5284. 10.1038/onc.2017.136 28504722

[B24] Choubey V. Zeb A. Kaasik A. (2021). Molecular mechanisms and regulation of mammalian mitophagy. Cells 11, 38. 10.3390/cells11010038 35011599 PMC8750762

[B25] Chu J. Y. K. Ou J. J. (2021). Autophagy in HCV replication and protein trafficking. Int. J. Mol. Sci. 22, 1089. 10.3390/ijms22031089 33499186 PMC7865906

[B26] Cianfanelli V. De Zio D. Di Bartolomeo S. Nazio F. Strappazzon F. Cecconi F. (2015). Ambra1 at a glance. J. Cell Sci. 128, 2003–2008. 10.1242/jcs.168153 26034061

[B27] Costa D. K. Huckestein B. R. Edmunds L. R. Petersen M. C. Nasiri A. Butrico G. M. (2016). Reduced intestinal lipid absorption and body weight-independent improvements in insulin sensitivity in high-fat diet-fed Park2 knockout mice. Am. J. Physiol. Endocrinol. Metab. 311, E105–E116. 10.1152/ajpendo.00042.2016 27166280 PMC4967148

[B28] Cover C. Mansouri A. Knight T. R. Bajt M. L. Lemasters J. J. Pessayre D. (2005). Peroxynitrite-induced mitochondrial and endonuclease-mediated nuclear DNA damage in acetaminophen hepatotoxicity. J. Pharmacol. Exp. Ther. 315, 879–887. 10.1124/jpet.105.088898 16081675

[B29] Cunningham C. C. Coleman W. B. Spach P. I. (1990). The effects of chronic ethanol consumption on hepatic mitochondrial energy metabolism. Alcohol Alcohol 25, 127–136. 10.1093/oxfordjournals.alcalc.a044987 2142884

[B30] Dahlin D. C. Miwa G. T. Lu A. Y. Nelson S. D. (1984). N-acetyl-p-benzoquinone imine: a cytochrome P-450-mediated oxidation product of acetaminophen. Proc. Natl. Acad. Sci. U. S. A. 81, 1327–1331. 10.1073/pnas.81.5.1327 6424115 PMC344826

[B31] Danpanichkul P. Suparan K. Ng C. H. Dejvajara D. Kongarin S. Panpradist N. (2024). Global and regional burden of alcohol-associated liver disease and alcohol use disorder in the elderly. JHEP Rep. 6, 101020. 10.1016/j.jhepr.2024.101020 38515553 PMC10956070

[B32] Deas E. Plun-Favreau H. Gandhi S. Desmond H. Kjaer S. Loh S. H. (2011). PINK1 cleavage at position A103 by the mitochondrial protease PARL. Hum. Mol. Genet. 20, 867–879. 10.1093/hmg/ddq526 21138942 PMC3033179

[B33] Ding W. X. Li M. Chen X. Ni H. M. Lin C. W. Gao W. (2010). Autophagy reduces acute ethanol-induced hepatotoxicity and steatosis in mice. Gastroenterology 139, 1740–1752. 10.1053/j.gastro.2010.07.041 20659474 PMC4129642

[B34] Ding W. X. Li M. Yin X. M. (2011). Selective taste of ethanol-induced autophagy for mitochondria and lipid droplets. Autophagy 7, 248–249. 10.4161/auto.7.2.14347 21150309 PMC3039771

[B35] Di Rita A. Peschiaroli A. P D. A. Strobbe D. Hu Z. Gruber J. (2018). HUWE1 E3 ligase promotes PINK1/PARKIN-independent mitophagy by regulating AMBRA1 activation *via* IKKα. Nat. Commun. 9, 3755. 10.1038/s41467-018-05722-3 30217973 PMC6138665

[B36] Du Q. Wu X. Ma K. Liu W. Liu P. Hayashi T. (2023). Silibinin alleviates ferroptosis of rat islet β cell INS-1 induced by the treatment with palmitic acid and high glucose through enhancing PINK1/parkin-mediated mitophagy. Arch. Biochem. Biophys. 743, 109644. 10.1016/j.abb.2023.109644 37245586

[B37] Eid N. Ito Y. Horibe A. Otsuki Y. (2016). Ethanol-induced mitophagy in liver is associated with activation of the PINK1-Parkin pathway triggered by oxidative DNA damage. Histol. Histopathol. 31, 1143–1159. 10.14670/HH-11-747 26935412

[B38] Eid N. Ito Y. Maemura K. Otsuki Y. (2013). Elevated autophagic sequestration of mitochondria and lipid droplets in steatotic hepatocytes of chronic ethanol-treated rats: an immunohistochemical and electron microscopic study. J. Mol. Histol. 44, 311–326. 10.1007/s10735-013-9483-x 23371376

[B39] Farré J. C. Krick R. Subramani S. Thumm M. (2009). Turnover of organelles by autophagy in yeast. Curr. Opin. Cell Biol. 21, 522–530. 10.1016/j.ceb.2009.04.015 19515549 PMC2725217

[B40] Fatima K. Mathew S. Suhail M. Ali A. Damanhouri G. Azhar E. (2014). Docking studies of Pakistani HCV NS3 helicase: a possible antiviral drug target. PLoS One 9, e106339. 10.1371/journal.pone.0106339 25188400 PMC4154687

[B41] Fisicaro P. Barili V. Montanini B. Acerbi G. Ferracin M. Guerrieri F. (2017). Targeting mitochondrial dysfunction can restore antiviral activity of exhausted HBV-Specific CD8 T cells in chronic hepatitis B. Nat. Med. 23, 327–336. 10.1038/nm.4275 28165481

[B42] Furuya N. Kakuta S. Sumiyoshi K. Ando M. Nonaka R. Suzuki A. (2018). NDP52 interacts with mitochondrial RNA poly(A) polymerase to promote mitophagy. EMBO Rep. 19, e46363. 10.15252/embr.201846363 30309841 PMC6280801

[B43] Ganesan P. Kulik L. M. (2023). Hepatocellular carcinoma: new developments. Clin. Liver Dis. 27, 85–102. 10.1016/j.cld.2022.08.004 36400469

[B44] Gao B. Bataller R. (2011). Alcoholic liver disease: pathogenesis and new therapeutic targets. Gastroenterology 141, 1572–1585. 10.1053/j.gastro.2011.09.002 21920463 PMC3214974

[B45] Gao F. Chen D. Si J. Hu Q. Qin Z. Fang M. (2015). The mitochondrial protein BNIP3L is the substrate of PARK2 and mediates mitophagy in PINK1/PARK2 pathway. Hum. Mol. Genet. 24, 2528–2538. 10.1093/hmg/ddv017 25612572

[B46] Gao H. Lv Y. Liu Y. Li J. Wang X. Zhou Z. (2019). Wolfberry-derived zeaxanthin dipalmitate attenuates ethanol-induced hepatic damage. Mol. Nutr. Food Res. 63, e1801339. 10.1002/mnfr.201801339 30938072

[B47] Gao Z. Yi W. Tang J. Sun Y. Huang J. Lan T. (2022). Urolithin A protects against acetaminophen-induced liver injury in mice *via* sustained activation of Nrf2. Int. J. Biol. Sci. 18, 2146–2162. 10.7150/ijbs.69116 35342347 PMC8935220

[B48] García-Ruiz C. Baulies A. Mari M. García-Rovés P. M. Fernandez-Checa J. C. (2013). Mitochondrial dysfunction in non-alcoholic fatty liver disease and insulin resistance: cause or consequence? Free Radic. Res. 47, 854–868. 10.3109/10715762.2013.830717 23915028

[B49] Geetha T. Wooten M. W. (2002). Structure and functional properties of the ubiquitin binding protein p62. FEBS Lett. 512, 19–24. 10.1016/s0014-5793(02)02286-x 11852044

[B50] Gegg M. E. Cooper J. M. Chau K. Y. Rojo M. Schapira A. H. Taanman J. W. (2010). Mitofusin 1 and mitofusin 2 are ubiquitinated in a PINK1/parkin-dependent manner upon induction of mitophagy. Hum. Mol. Genet. 19, 4861–4870. 10.1093/hmg/ddq419 20871098 PMC3583518

[B51] Geisler S. Holmström K. M. Skujat D. Fiesel F. C. Rothfuss O. C. Kahle P. J. (2010). PINK1/Parkin-mediated mitophagy is dependent on VDAC1 and p62/SQSTM1. Nat. Cell Biol. 12, 119–131. 10.1038/ncb2012 20098416

[B52] Glick D. Zhang W. Beaton M. Marsboom G. Gruber M. Simon M. C. (2012). BNip3 regulates mitochondrial function and lipid metabolism in the liver. Mol. Cell Biol. 32, 2570–2584. 10.1128/MCB.00167-12 22547685 PMC3434502

[B53] Gomez-Suaga P. Paillusson S. Stoica R. Noble W. Hanger D. P. Miller C. C. J. (2017). The ER-Mitochondria tethering complex VAPB-PTPIP51 regulates autophagy. Curr. Biol. 27, 371–385. 10.1016/j.cub.2016.12.038 28132811 PMC5300905

[B54] Gong L. L. Yang S. Zhang W. Han F. F. Lv Y. L. Wan Z. R. (2018). Akebia saponin D alleviates hepatic steatosis through BNip3 induced mitophagy. J. Pharmacol. Sci. 136, 189–195. 10.1016/j.jphs.2017.11.007 29609842

[B55] Greene A. W. Grenier K. Aguileta M. A. Muise S. Farazifard R. Haque M. E. (2012). Mitochondrial processing peptidase regulates PINK1 processing, import and parkin recruitment. EMBO Rep. 13, 378–385. 10.1038/embor.2012.14 22354088 PMC3321149

[B56] Guerrieri F. Belloni L. Pediconi N. Levrero M. (2013). Molecular mechanisms of HBV-Associated hepatocarcinogenesis. Semin. Liver Dis. 33, 147–156. 10.1055/s-0033-1345721 23749671

[B57] Han D. Johnson H. S. Rao M. P. Martin G. Sancheti H. Silkwood K. H. (2017). Mitochondrial remodeling in the liver following chronic alcohol feeding to rats. Free Radic. Biol. Med. 102, 100–110. 10.1016/j.freeradbiomed.2016.11.020 27867097 PMC5209270

[B58] Hanna R. A. Quinsay M. N. Orogo A. M. Giang K. Rikka S. Gustafsson Å B. (2012). Microtubule-associated protein 1 light chain 3 (LC3) interacts with Bnip3 protein to selectively remove endoplasmic reticulum and mitochondria *via* autophagy. J. Biol. Chem. 287, 19094–19104. 10.1074/jbc.M111.322933 22505714 PMC3365942

[B59] Hara Y. Yanatori I. Ikeda M. Kiyokage E. Nishina S. Tomiyama Y. (2014). Hepatitis C virus core protein suppresses mitophagy by interacting with parkin in the context of mitochondrial depolarization. Am. J. Pathol. 184, 3026–3039. 10.1016/j.ajpath.2014.07.024 25244949

[B60] Hernando-Rodríguez B. Artal-Sanz M. (2018). Mitochondrial quality control mechanisms and the PHB (prohibitin) complex. Cells 7. 10.3390/cells7120238 PMC6315423 30501123

[B61] Houghton M. (2019). Hepatitis C virus: 30 years after its discovery. Cold Spring Harb. Perspect. Med. 9, a037069. 10.1101/cshperspect.a037069 31501269 PMC6886456

[B62] Huang T. Xu T. Wang Y. Zhou Y. Yu D. Wang Z. (2021). Cannabidiol inhibits human glioma by induction of lethal mitophagy through activating TRPV4. Autophagy 17, 3592–3606. 10.1080/15548627.2021.1885203 33629929 PMC8632311

[B63] Huang X. Y. Li D. Chen Z. X. Huang Y. H. Gao W. Y. Zheng B. Y. (2018). Hepatitis B virus X protein elevates Parkin-mediated mitophagy through lon peptidase in starvation. Exp. Cell Res. 368, 75–83. 10.1016/j.yexcr.2018.04.016 29689279

[B64] Ichimura Y. Kumanomidou T. Sou Y. S. Mizushima T. Ezaki J. Ueno T. (2008). Structural basis for sorting mechanism of p62 in selective autophagy. J. Biol. Chem. 283, 22847–22857. 10.1074/jbc.M802182200 18524774

[B65] Jaeschke H. Mcgill M. R. Ramachandran A. (2012). Oxidant stress, mitochondria, and cell death mechanisms in drug-induced liver injury: lessons learned from acetaminophen hepatotoxicity. Drug Metab. Rev. 44, 88–106. 10.3109/03602532.2011.602688 22229890 PMC5319847

[B66] Jassey A. Liu C. H. Changou C. A. Richardson C. D. Hsu H. Y. Lin L. T. (2019). Hepatitis C virus non-structural protein 5A (NS5A) disrupts mitochondrial dynamics and induces mitophagy. Cells 8, 290. 10.3390/cells8040290 30934919 PMC6523690

[B67] Jin S. M. Lazarou M. Wang C. Kane L. A. Narendra D. P. Youle R. J. (2010). Mitochondrial membrane potential regulates PINK1 import and proteolytic destabilization by PARL. J. Cell Biol. 191, 933–942. 10.1083/jcb.201008084 21115803 PMC2995166

[B68] Johansen T. Lamark T. (2011). Selective autophagy mediated by autophagic adapter proteins. Autophagy 7, 279–296. 10.4161/auto.7.3.14487 21189453 PMC3060413

[B69] Jollow D. (2024). Acetaminophen-induced hepatic necrosis: a reminiscence. Drug Metab. Dispos. 52, 707–711. 10.1124/dmd.123.001278 37793785

[B70] Kang X. Wang H. Li Y. Xiao Y. Zhao L. Zhang T. (2019). Alantolactone induces apoptosis through ROS-Mediated AKT pathway and inhibition of PINK1-mediated mitophagy in human HepG2 cells. Artif. Cells Nanomed Biotechnol. 47, 1961–1970. 10.1080/21691401.2019.1593854 31116036

[B71] Kawajiri S. Saiki S. Sato S. Sato F. Hatano T. Eguchi H. (2010). PINK1 is recruited to mitochondria with parkin and associates with LC3 in mitophagy. FEBS Lett. 584, 1073–1079. 10.1016/j.febslet.2010.02.016 20153330

[B72] Khambu B. Wang L. Zhang H. Yin X. M. (2017). The activation and function of autophagy in alcoholic liver disease. Curr. Mol. Pharmacol. 10, 165–171. 10.2174/1874467208666150817112654 26278385 PMC5585070

[B73] Khan M. Imam H. Siddiqui A. (2018). Subversion of cellular autophagy during virus infection: insights from hepatitis B and hepatitis C viruses. Liver Res. 2, 146–156. 10.1016/j.livres.2018.09.002 31803515 PMC6892584

[B74] Kim K. Y. Sack M. N. (2012). Parkin in the regulation of fat uptake and mitochondrial biology: emerging links in the pathophysiology of parkinson's disease. Curr. Opin. Lipidol. 23, 201–205. 10.1097/MOL.0b013e328352dc5d 22488424 PMC4151552

[B75] Kim K. Y. Stevens M. V. Akter M. H. Rusk S. E. Huang R. J. Cohen A. (2011). Parkin is a lipid-responsive regulator of fat uptake in mice and mutant human cells. J. Clin. Invest 121, 3701–3712. 10.1172/JCI44736 21865652 PMC3171101

[B76] Kim S. J. Jang J. Y. Kim E. J. Cho E. K. Ahn D. G. Kim C. (2017). Ginsenoside Rg3 restores hepatitis C virus-induced aberrant mitochondrial dynamics and inhibits virus propagation. Hepatology 66, 758–771. 10.1002/hep.29177 28329914 PMC5755973

[B77] Kim S. J. Khan M. Quan J. Till A. Subramani S. Siddiqui A. (2013a). Hepatitis B virus disrupts mitochondrial dynamics: induces fission and mitophagy to attenuate apoptosis. PLoS Pathog. 9, e1003722. 10.1371/journal.ppat.1003722 24339771 PMC3855539

[B78] Kim S. J. Syed G. H. Khan M. Chiu W. W. Sohail M. A. Gish R. G. (2014). Hepatitis C virus triggers mitochondrial fission and attenuates apoptosis to promote viral persistence. Proc. Natl. Acad. Sci. U. S. A. 111, 6413–6418. 10.1073/pnas.1321114111 24733894 PMC4035934

[B79] Kim S. J. Syed G. H. Siddiqui A. (2013b). Hepatitis C virus induces the mitochondrial translocation of parkin and subsequent mitophagy. PLoS Pathog. 9, e1003285. 10.1371/journal.ppat.1003285 23555273 PMC3610669

[B80] Kirkin V. Mcewan D. G. Novak I. Dikic I. (2009). A role for ubiquitin in selective autophagy. Mol. Cell 34, 259–269. 10.1016/j.molcel.2009.04.026 19450525

[B81] Koentjoro B. Park J. S. Sue C. M. (2017). Nix restores mitophagy and mitochondrial function to protect against PINK1/Parkin-related parkinson's disease. Sci. Rep. 7, 44373. 10.1038/srep44373 28281653 PMC5345073

[B82] Komatsu M. Ichimura Y. (2010). Physiological significance of selective degradation of p62 by autophagy. FEBS Lett. 584, 1374–1378. 10.1016/j.febslet.2010.02.017 20153326

[B83] Kon K. Kim J. S. Jaeschke H. Lemasters J. J. (2004). Mitochondrial permeability transition in acetaminophen-induced necrosis and apoptosis of cultured mouse hepatocytes. Hepatology 40, 1170–1179. 10.1002/hep.20437 15486922

[B84] Koyano F. Okatsu K. Kosako H. Tamura Y. Go E. Kimura M. (2014). Ubiquitin is phosphorylated by PINK1 to activate parkin. Nature 510, 162–166. 10.1038/nature13392 24784582

[B85] Kubli D. A. Quinsay M. N. Huang C. Lee Y. Gustafsson A. B. (2008). Bnip3 functions as a mitochondrial sensor of oxidative stress during myocardial ischemia and reperfusion. Am. J. Physiol. Heart Circ. Physiol. 295, H2025–H2031. 10.1152/ajpheart.00552.2008 18790835 PMC2614576

[B86] Lanini S. Ustianowski A. Pisapia R. Zumla A. Ippolito G. (2019). Viral hepatitis: etiology, epidemiology, transmission, diagnostics, treatment, and prevention. Infect. Dis. Clin. North Am. 33, 1045–1062. 10.1016/j.idc.2019.08.004 31668190

[B87] Larson A. M. Polson J. Fontana R. J. Davern T. J. Lalani E. Hynan L. S. (2005). Acetaminophen-induced acute liver failure: results of a United States multicenter, prospective study. Hepatology 42, 1364–1372. 10.1002/hep.20948 16317692

[B88] Lee J. Park J. S. Roh Y. S. (2019). Molecular insights into the role of mitochondria in non-alcoholic fatty liver disease. Arch. Pharm. Res. 42, 935–946. 10.1007/s12272-019-01178-1 31571145

[B89] Lee W. M. (2013). Drug-induced acute liver failure. Clin. Liver Dis. 17, 575–586. viii. 10.1016/j.cld.2013.07.001 24099019 PMC3838908

[B90] Lemasters J. J. (2005). Selective mitochondrial autophagy, or mitophagy, as a targeted defense against oxidative stress, mitochondrial dysfunction, and aging. Rejuvenation Res. 8, 3–5. 10.1089/rej.2005.8.3 15798367

[B91] Lemasters J. J. Nieminen A. L. Qian T. Trost L. C. Elmore S. P. Nishimura Y. (1998). The mitochondrial permeability transition in cell death: a common mechanism in necrosis, apoptosis and autophagy. Biochim. Biophys. Acta 1366, 177–196. 10.1016/s0005-2728(98)00112-1 9714796

[B92] Lemasters J. J. Zhong Z. (2018). Mitophagy in hepatocytes: types, initiators and role in adaptive ethanol metabolism☆. Liver Res. 2 **,** 125–132. 10.1016/j.livres.2018.09.005 31157120 PMC6541449

[B93] Levine B. Kroemer G. (2008). Autophagy in the pathogenesis of disease. Cell 132, 27–42. 10.1016/j.cell.2007.12.018 18191218 PMC2696814

[B94] Li G. Li J. Shao R. Zhao J. Chen M. (2021a). FUNDC1: a promising mitophagy regulator at the mitochondria-associated membrane for cardiovascular diseases. Front. Cell Dev. Biol. 9, 788634. 10.3389/fcell.2021.788634 35096821 PMC8797154

[B95] Li R. Xin T. Li D. Wang C. Zhu H. Zhou H. (2018). Therapeutic effect of sirtuin 3 on ameliorating nonalcoholic fatty liver disease: the role of the ERK-CREB pathway and Bnip3-mediated mitophagy. Redox Biol. 18, 229–243. 10.1016/j.redox.2018.07.011 30056271 PMC6079484

[B96] Li S. Zhang J. Liu C. Wang Q. Yan J. Hui L. (2021b). The role of mitophagy in regulating cell death. Oxid. Med. Cell Longev. 2021, 6617256. 10.1155/2021/6617256 34113420 PMC8154277

[B97] Li W. Li Y. Siraj S. Jin H. Fan Y. Yang X. (2019). FUN14 domain-containing 1-Mediated mitophagy suppresses hepatocarcinogenesis by inhibition of inflammasome activation in mice. Hepatology 69, 604–621. 10.1002/hep.30191 30053328

[B98] Li X. Shi Z. Zhu Y. Shen T. Wang H. Shui G. (2020). Cyanidin-3-O-glucoside improves non-alcoholic fatty liver disease by promoting PINK1-mediated mitophagy in mice. Br. J. Pharmacol. 177, 3591–3607. 10.1111/bph.15083 32343398 PMC7348088

[B99] Linden M. A. Lopez K. T. Fletcher J. A. Morris E. M. Meers G. M. Siddique S. (2015). Combining metformin therapy with caloric restriction for the management of type 2 diabetes and nonalcoholic fatty liver disease in Obese rats. Appl. Physiol. Nutr. Metab. 40, 1038–1047. 10.1139/apnm-2015-0236 26394261 PMC4713237

[B100] Liu J. Jiang S. Zhao Y. Sun Q. Zhang J. Shen D. (2018a). Geranylgeranyl diphosphate synthase (GGPPS) regulates non-alcoholic fatty liver disease (NAFLD)-Fibrosis progression by determining hepatic glucose/fatty acid preference under high-fat diet conditions. J. Pathol. 246, 277–288. 10.1002/path.5131 29971772

[B101] Liu K. Lee J. Kim J. Y. Wang L. Tian Y. Chan S. T. (2017a). Mitophagy controls the activities of tumor suppressor p53 to regulate hepatic cancer stem cells. Mol. Cell 68, 281–292.e5. 10.1016/j.molcel.2017.09.022 29033320 PMC5687282

[B102] Liu L. Feng D. Chen G. Chen M. Zheng Q. Song P. (2012). Mitochondrial outer-membrane protein FUNDC1 mediates hypoxia-induced mitophagy in Mammalian cells. Nat. Cell Biol. 14, 177–185. 10.1038/ncb2422 22267086

[B103] Liu P. Lin H. Xu Y. Zhou F. Wang J. Liu J. (2018b). Frataxin-mediated PINK1-Parkin-Dependent mitophagy in hepatic steatosis: the protective effects of quercetin. Mol. Nutr. Food Res. 62, e1800164. 10.1002/mnfr.201800164 29935106

[B104] Liu X. Hussain R. Mehmood K. Tang Z. Zhang H. Li Y. (2022). Mitochondrial-endoplasmic reticulum communication-mediated oxidative stress and autophagy. Biomed. Res. Int. 2022, 6459585. 10.1155/2022/6459585 36164446 PMC9509228

[B105] Liu Z. Ren B. Wang Y. Zou C. Qiao Q. Diao Z. (2017b). Sesamol induces human hepatocellular carcinoma cells apoptosis by impairing mitochondrial function and suppressing autophagy. Sci. Rep. 7, 45728. 10.1038/srep45728 28374807 PMC5379556

[B106] Lu X. Xuan W. Li J. Yao H. Huang C. Li J. (2021). AMPK protects against alcohol-induced liver injury through UQCRC2 to up-regulate mitophagy. Autophagy 17, 3622–3643. 10.1080/15548627.2021.1886829 33719895 PMC8632272

[B107] Lumeng L. Crabb D. W. (2000). Alcoholic liver disease. Curr. Opin. Gastroenterol. 16, 208–218. 10.1097/00001574-200005000-00003 17023878

[B108] Ma G. D. Liu Y. H. Zhang Q. L. Zhang B. G. Zhao N. Wang Q. L. (2014). Pre-endurance training prevents acute alcoholic liver injury in rats through the regulation of damaged mitochondria accumulation and mitophagy balance. Hepatol. Int. 8, 425–435. 10.1007/s12072-014-9529-5 26202644

[B109] Mansouri A. Gaou I. De Kerguenec C. Amsellem S. Haouzi D. Berson A. (1999). An alcoholic binge causes massive degradation of hepatic mitochondrial DNA in mice. Gastroenterology 117, 181–190. 10.1016/s0016-5085(99)70566-4 10381926

[B110] Matsuda N. Sato S. Shiba K. Okatsu K. Saisho K. Gautier C. A. (2010). PINK1 stabilized by mitochondrial depolarization recruits parkin to damaged mitochondria and activates latent parkin for mitophagy. J. Cell Biol. 189, 211–221. 10.1083/jcb.200910140 20404107 PMC2856912

[B111] Mcgill M. R. Jaeschke H. (2013). Metabolism and disposition of acetaminophen: recent advances in relation to hepatotoxicity and diagnosis. Pharm. Res. 30, 2174–2187. 10.1007/s11095-013-1007-6 23462933 PMC3709007

[B112] Mcwilliams T. G. Prescott A. R. Allen G. F. Tamjar J. Munson M. J. Thomson C. (2016). mito-QC illuminates mitophagy and mitochondrial architecture *in vivo* . J. Cell Biol. 214, 333–345. 10.1083/jcb.201603039 27458135 PMC4970326

[B113] Meissner C. Lorenz H. Weihofen A. Selkoe D. J. Lemberg M. K. (2011). The mitochondrial intramembrane protease PARL cleaves human Pink1 to regulate Pink1 trafficking. J. Neurochem. 117, 856–867. 10.1111/j.1471-4159.2011.07253.x 21426348

[B114] Meyer N. Zielke S. Michaelis J. B. Linder B. Warnsmann V. Rakel S. (2018). AT 101 induces early mitochondrial dysfunction and HMOX1 (heme oxygenase 1) to trigger mitophagic cell death in glioma cells. Autophagy 14, 1693–1709. 10.1080/15548627.2018.1476812 29938581 PMC6135628

[B115] Montali I. Ceccatelli Berti C. Morselli M. Acerbi G. Barili V. Pedrazzi G. (2023). Deregulated intracellular pathways define novel molecular targets for HBV-Specific CD8 T cell reconstitution in chronic hepatitis B. J. Hepatol. 79, 50–60. 10.1016/j.jhep.2023.02.035 36893853

[B116] Moradpour D. Penin F. Rice C. M. (2007). Replication of hepatitis C virus. Nat. Rev. Microbiol. 5, 453–463. 10.1038/nrmicro1645 17487147

[B117] Moscat J. Diaz-Meco M. T. Wooten M. W. (2007). Signal integration and diversification through the p62 scaffold protein. Trends Biochem. Sci. 32, 95–100. 10.1016/j.tibs.2006.12.002 17174552

[B118] Murakawa T. Ito J. Rusu M. C. Taneike M. Omiya S. Moncayo-Arlandi J. (2024). AMPK regulates Bcl2-L-13-mediated mitophagy induction for cardioprotection. Cell Rep. 43, 115001. 10.1016/j.celrep.2024.115001 39580803 PMC11672683

[B119] Murakawa T. Okamoto K. Omiya S. Taneike M. Yamaguchi O. Otsu K. (2019). A mammalian mitophagy receptor, Bcl2-L-13, recruits the ULK1 complex to induce mitophagy. Cell Rep. 26, 338–345.e6. 10.1016/j.celrep.2018.12.050 30625316 PMC6326162

[B120] Murakawa T. Yamaguchi O. Hashimoto A. Hikoso S. Takeda T. Oka T. (2015). Bcl-2-like protein 13 is a Mammalian Atg32 homologue that mediates mitophagy and mitochondrial fragmentation. Nat. Commun. 6, 7527. 10.1038/ncomms8527 26146385 PMC4501433

[B121] Nakahira K. Haspel J. A. Rathinam V. A. Lee S. J. Dolinay T. Lam H. C. (2011). Autophagy proteins regulate innate immune responses by inhibiting the release of mitochondrial DNA mediated by the NALP3 inflammasome. Nat. Immunol. 12, 222–230. 10.1038/ni.1980 21151103 PMC3079381

[B122] Narendra D. Tanaka A. Suen D. F. Youle R. J. (2008). Parkin is recruited selectively to impaired mitochondria and promotes their autophagy. J. Cell Biol. 183, 795–803. 10.1083/jcb.200809125 19029340 PMC2592826

[B123] Narendra D. P. Jin S. M. Tanaka A. Suen D. F. Gautier C. A. Shen J. (2010). PINK1 is selectively stabilized on impaired mitochondria to activate parkin. PLoS Biol. 8, e1000298. 10.1371/journal.pbio.1000298 20126261 PMC2811155

[B124] Nazio F. Strappazzon F. Antonioli M. Bielli P. Cianfanelli V. Bordi M. (2013). mTOR inhibits autophagy by controlling ULK1 ubiquitylation, self-association and function through AMBRA1 and TRAF6. Nat. Cell Biol. 15, 406–416. 10.1038/ncb2708 23524951

[B125] Neuveut C. Wei Y. Buendia M. A. (2010). Mechanisms of HBV-Related hepatocarcinogenesis. J. Hepatol. 52, 594–604. 10.1016/j.jhep.2009.10.033 20185200

[B126] Ni H. M. Bockus A. Boggess N. Jaeschke H. Ding W. X. (2012). Activation of autophagy protects against acetaminophen-induced hepatotoxicity. Hepatology 55, 222–232. 10.1002/hep.24690 21932416 PMC3245329

[B127] Nickel A. Kohlhaas M. Maack C. (2014). Mitochondrial reactive oxygen species production and elimination. J. Mol. Cell Cardiol. 73, 26–33. 10.1016/j.yjmcc.2014.03.011 24657720

[B128] Novak I. Kirkin V. Mcewan D. G. Zhang J. Wild P. Rozenknop A. (2010). Nix is a selective autophagy receptor for mitochondrial clearance. EMBO Rep. 11, 45–51. 10.1038/embor.2009.256 20010802 PMC2816619

[B129] Padman B. S. Nguyen T. N. Uoselis L. Skulsuppaisarn M. Nguyen L. K. Lazarou M. (2019). LC3/GABARAPs drive ubiquitin-independent recruitment of optineurin and NDP52 to amplify mitophagy. Nat. Commun. 10, 408. 10.1038/s41467-019-08335-6 30679426 PMC6345886

[B130] Palma E. Ma X. Riva A. Iansante V. Dhawan A. Wang S. (2019). Dynamin-1-Like protein inhibition drives megamitochondria formation as an adaptive response in alcohol-induced hepatotoxicity. Am. J. Pathol. 189, 580–589. 10.1016/j.ajpath.2018.11.008 30553835 PMC6436109

[B131] Pankiv S. Clausen T. H. Lamark T. Brech A. Bruun J. A. Outzen H. (2007). p62/SQSTM1 binds directly to Atg8/LC3 to facilitate degradation of ubiquitinated protein aggregates by autophagy. J. Biol. Chem. 282, 24131–24145. 10.1074/jbc.M702824200 17580304

[B132] Pankiv S. Lamark T. Bruun J. A. Øvervatn A. Bjørkøy G. Johansen T. (2010). Nucleocytoplasmic shuttling of p62/SQSTM1 and its role in recruitment of nuclear polyubiquitinated proteins to promyelocytic leukemia bodies. J. Biol. Chem. 285, 5941–5953. 10.1074/jbc.M109.039925 20018885 PMC2820819

[B133] Pessayre D. Fromenty B. Berson A. Robin M. A. Lettéron P. Moreau R. (2012). Central role of mitochondria in drug-induced liver injury. Drug Metab. Rev. 44, 34–87. 10.3109/03602532.2011.604086 21892896

[B134] Pickles S. Vigié P. Youle R. J. (2018). Mitophagy and quality control mechanisms in mitochondrial maintenance. Curr. Biol. 28, R170–r185. 10.1016/j.cub.2018.01.004 29462587 PMC7255410

[B135] Potter W. Z. Davis D. C. Mitchell J. R. Jollow D. J. Gillette J. R. Brodie B. B. (1973). Acetaminophen-induced hepatic necrosis. 3. Cytochrome P-450-mediated covalent binding *in vitro* . J. Pharmacol. Exp. Ther. 187, 203–210. 10.1016/s0022-3565(25)29665-3 4147720

[B136] Qian H. Chao X. Ding W. X. (2018). A PINK1-mediated mitophagy pathway decides the fate of tumors-to be benign or malignant? Autophagy 14, 563–566. 10.1080/15548627.2018.1425057 29313453 PMC5959334

[B137] Qian Z. Liang J. Huang R. Song W. Ying J. Bi X. (2024). HBV integrations reshaping genomic structures promote hepatocellular carcinoma. Gut 73, 1169–1182. 10.1136/gutjnl-2023-330414 38395437 PMC11187386

[B138] Rahmani Z. Huh K. W. Lasher R. Siddiqui A. (2000). Hepatitis B virus X protein colocalizes to mitochondria with a human voltage-dependent anion channel, HVDAC3, and alters its transmembrane potential. J. Virol. 74, 2840–2846. 10.1128/jvi.74.6.2840-2846.2000 10684300 PMC111774

[B139] Ramachandran A. Jaeschke H. (2018). Acetaminophen toxicity: novel insights into mechanisms and future perspectives. Gene Expr. 18, 19–30. 10.3727/105221617X15084371374138 29054140 PMC5885144

[B140] Ramachandran A. Lebofsky M. Baines C. P. Lemasters J. J. Jaeschke H. (2011). Cyclophilin D deficiency protects against acetaminophen-induced oxidant stress and liver injury. Free Radic. Res. 45, 156–164. 10.3109/10715762.2010.520319 20942566 PMC3899524

[B141] Ray R. Chen G. Vande Velde C. Cizeau J. Park J. H. Reed J. C. (2000). BNIP3 heterodimerizes with Bcl-2/Bcl-X(L) and induces cell death independent of a Bcl-2 homology 3 (BH3) domain at both mitochondrial and nonmitochondrial sites. J. Biol. Chem. 275, 1439–1448. 10.1074/jbc.275.2.1439 10625696

[B142] Rogov V. V. Suzuki H. Marinković M. Lang V. Kato R. Kawasaki M. (2017). Phosphorylation of the mitochondrial autophagy receptor nix enhances its interaction with LC3 proteins. Sci. Rep. 7, 1131. 10.1038/s41598-017-01258-6 28442745 PMC5430633

[B143] Rojansky R. Cha M. Y. Chan D. C. (2016). Elimination of paternal mitochondria in mouse embryos occurs through autophagic degradation dependent on PARKIN and MUL1. Elife 5, e17896. 10.7554/eLife.17896 27852436 PMC5127638

[B144] Ryan T. A. Tumbarello D. A. (2018). Optineurin: a coordinator of membrane-associated cargo trafficking and autophagy. Front. Immunol. 9, 1024. 10.3389/fimmu.2018.01024 29867991 PMC5962687

[B145] Sandoval H. Thiagarajan P. Dasgupta S. K. Schumacher A. Prchal J. T. Chen M. (2008). Essential role for nix in autophagic maturation of erythroid cells. Nature 454, 232–235. 10.1038/nature07006 18454133 PMC2570948

[B146] Schieber M. Chandel N. S. (2014). ROS function in redox signaling and oxidative stress. Curr. Biol. 24, R453–R462. 10.1016/j.cub.2014.03.034 24845678 PMC4055301

[B147] Seibenhener M. L. Geetha T. Wooten M. W. (2007). Sequestosome 1/p62--more than just a scaffold. FEBS Lett. 581, 175–179. 10.1016/j.febslet.2006.12.027 17188686 PMC1850379

[B148] Severi T. Van Malenstein H. Verslype C. Van Pelt J. F. (2010). Tumor initiation and progression in hepatocellular carcinoma: risk factors, classification, and therapeutic targets. Acta Pharmacol. Sin. 31, 1409–1420. 10.1038/aps.2010.142 20953207 PMC4003336

[B149] Shaid S. Brandts C. H. Serve H. Dikic I. (2013). Ubiquitination and selective autophagy. Cell Death Differ. 20, 21–30. 10.1038/cdd.2012.72 22722335 PMC3524631

[B150] Shan S. Shen Z. Zhang C. Kou R. Xie K. Song F. (2019). Mitophagy protects against acetaminophen-induced acute liver injury in mice through inhibiting NLRP3 inflammasome activation. Biochem. Pharmacol. 169, 113643. 10.1016/j.bcp.2019.113643 31542387

[B151] Shao N. Yu X. Y. Ma X. F. Lin W. J. Hao M. Kuang H. Y. (2018). Exenatide delays the progression of nonalcoholic fatty liver disease in C57BL/6 mice, which may involve inhibition of the NLRP3 inflammasome through the mitophagy pathway. Gastroenterol. Res. Pract. 2018, 1864307. 10.1155/2018/1864307 29849583 PMC5925008

[B152] Shi C. Cai Y. Li Y. Li Y. Hu N. Ma S. (2018). Yap promotes hepatocellular carcinoma metastasis and mobilization *via* governing cofilin/F-actin/lamellipodium axis by regulation of JNK/Bnip3/SERCA/CaMKII pathways. Redox Biol. 14, 59–71. 10.1016/j.redox.2017.08.013 28869833 PMC5582718

[B153] Shi R. Y. Zhu S. H. Li V. Gibson S. B. Xu X. S. Kong J. M. (2014). BNIP3 interacting with LC3 triggers excessive mitophagy in delayed neuronal death in stroke. CNS Neurosci. Ther. 20, 1045–1055. 10.1111/cns.12325 25230377 PMC6492992

[B154] Shin J. (1998). P62 and the sequestosome, a novel mechanism for protein metabolism. Arch. Pharm. Res. 21, 629–633. 10.1007/BF02976748 9868528

[B155] Sia D. Villanueva A. Friedman S. L. Llovet J. M. (2017). Liver cancer cell of origin, molecular class, and effects on patient prognosis. Gastroenterology 152, 745–761. 10.1053/j.gastro.2016.11.048 28043904 PMC12160040

[B156] Simões I. C. M. Fontes A. Pinton P. Zischka H. Wieckowski M. R. (2018). Mitochondria in non-alcoholic fatty liver disease. Int. J. Biochem. Cell Biol. 95, 93–99. 10.1016/j.biocel.2017.12.019 29288054

[B157] Simula M. P. De Re V. (2010). Hepatitis C virus-induced oxidative stress and mitochondrial dysfunction: a focus on recent advances in proteomics. Proteomics Clin. Appl. 4, 782–793. 10.1002/prca.201000049 21137022

[B158] Sir D. Tian Y. Chen W. L. Ann D. K. Yen T. S. Ou J. H. (2010). The early autophagic pathway is activated by hepatitis B virus and required for viral DNA replication. Proc. Natl. Acad. Sci. U. S. A. 107, 4383–4388. 10.1073/pnas.0911373107 20142477 PMC2840127

[B159] Strappazzon F. Nazio F. Corrado M. Cianfanelli V. Romagnoli A. Fimia G. M. (2015). AMBRA1 is able to induce mitophagy *via* LC3 binding, regardless of PARKIN and p62/SQSTM1. Cell Death Differ. 22, 517. 10.1038/cdd.2014.190 25661525 PMC4326578

[B160] Tanaka A. Cleland M. M. Xu S. Narendra D. P. Suen D. F. Karbowski M. (2010). Proteasome and p97 mediate mitophagy and degradation of mitofusins induced by parkin. J. Cell Biol. 191, 1367–1380. 10.1083/jcb.201007013 21173115 PMC3010068

[B161] Tang Y. Gao C. Xing M. Li Y. Zhu L. Wang D. (2012). Quercetin prevents ethanol-induced dyslipidemia and mitochondrial oxidative damage. Food Chem. Toxicol. 50, 1194–1200. 10.1016/j.fct.2012.02.008 22365892

[B162] Thomas K. J. Mccoy M. K. Blackinton J. Beilina A. Van Der Brug M. Sandebring A. (2011). DJ-1 acts in parallel to the PINK1/parkin pathway to control mitochondrial function and autophagy. Hum. Mol. Genet. 20, 40–50. 10.1093/hmg/ddq430 20940149 PMC3000675

[B163] Thomes P. G. Ehlers R. A. Trambly C. S. Clemens D. L. Fox H. S. Tuma D. J. (2013). Multilevel regulation of autophagosome content by ethanol oxidation in HepG2 cells. Autophagy 9, 63–73. 10.4161/auto.22490 23090141 PMC3542219

[B164] Thurston T. L. Ryzhakov G. Bloor S. Von Muhlinen N. Randow F. (2009). The TBK1 adaptor and autophagy receptor NDP52 restricts the proliferation of ubiquitin-coated bacteria. Nat. Immunol. 10, 1215–1221. 10.1038/ni.1800 19820708

[B165] Tong J. Lan X. T. Zhang Z. Liu Y. Sun D. Y. Wang X. J. (2023). Ferroptosis inhibitor liproxstatin-1 alleviates metabolic dysfunction-associated fatty liver disease in mice: potential involvement of PANoptosis. Acta Pharmacol. Sin. 44, 1014–1028. 10.1038/s41401-022-01010-5 36323829 PMC10104837

[B166] Torresi J. Tran B. M. Christiansen D. Earnest-Silveira L. Schwab R. H. M. Vincan E. (2019). HBV-Related hepatocarcinogenesis: the role of signalling pathways and innovative *ex vivo* research models. BMC Cancer 19, 707. 10.1186/s12885-019-5916-6 31319796 PMC6637598

[B167] Tsukuda S. Watashi K. (2020). Hepatitis B virus biology and life cycle. Antivir. Res. 182, 104925. 10.1016/j.antiviral.2020.104925 32866519

[B168] Uchida T. Kronborg I. Peters R. L. (1984). Giant mitochondria in the alcoholic liver diseases--their identification, frequency and pathologic significance. Liver 4, 29–38. 10.1111/j.1600-0676.1984.tb00904.x 6700382

[B169] Van Humbeeck C. Cornelissen T. Hofkens H. Mandemakers W. Gevaert K. De Strooper B. (2011). Parkin interacts with Ambra1 to induce mitophagy. J. Neurosci. 31, 10249–10261. 10.1523/JNEUROSCI.1917-11.2011 21753002 PMC6623066

[B170] Vera-Ramirez L. Sanchez-Rovira P. Ramirez-Tortosa M. C. Ramirez-Tortosa C. L. Granados-Principal S. Lorente J. A. (2011). Free radicals in breast carcinogenesis, breast cancer progression and cancer stem cells. Biological bases to develop oxidative-based therapies. Crit. Rev. Oncol. Hematol. 80, 347–368. 10.1016/j.critrevonc.2011.01.004 21288735

[B171] Vives-Bauza C. Zhou C. Huang Y. Cui M. De Vries R. L. Kim J. (2010). PINK1-dependent recruitment of parkin to mitochondria in mitophagy. Proc. Natl. Acad. Sci. U. S. A. 107, 378–383. 10.1073/pnas.0911187107 19966284 PMC2806779

[B172] Von Muhlinen N. Akutsu M. Ravenhill B. J. Foeglein Á. Bloor S. Rutherford T. J. (2012). LC3C, bound selectively by a noncanonical LIR motif in NDP52, is required for antibacterial autophagy. Mol. Cell 48, 329–342. 10.1016/j.molcel.2012.08.024 23022382 PMC3510444

[B173] Wang C. Wang Y. (2023). The role and mechanism of action of mitophagy in various liver diseases. Antioxid. Redox Signal 38, 529–549. 10.1089/ars.2022.0114 36017629

[B174] Wang H. Ni H. M. Chao X. Ma X. Rodriguez Y. A. Chavan H. (2019). Double deletion of PINK1 and parkin impairs hepatic mitophagy and exacerbates acetaminophen-induced liver injury in mice. Redox Biol. 22, 101148. 10.1016/j.redox.2019.101148 30818124 PMC6395945

[B175] Wang L. Liu X. Nie J. Zhang J. Kimball S. R. Zhang H. (2015). ALCAT1 controls mitochondrial etiology of fatty liver diseases, linking defective mitophagy to steatosis. Hepatology 61, 486–496. 10.1002/hep.27420 25203315 PMC4303512

[B176] Wang X. Winter D. Ashrafi G. Schlehe J. Wong Y. L. Selkoe D. (2011). PINK1 and parkin target miro for phosphorylation and degradation to arrest mitochondrial motility. Cell 147, 893–906. 10.1016/j.cell.2011.10.018 22078885 PMC3261796

[B177] Wang Y. Wang Z. Sun J. Qian Y. (2021). Identification of HCC subtypes with different prognosis and metabolic patterns based on mitophagy. Front. Cell Dev. Biol. 9, 799507. 10.3389/fcell.2021.799507 34977039 PMC8716756

[B178] Wei Y. Chiang W. C. Sumpter R. Jr. Mishra P. Levine B. (2017). Prohibitin 2 is an inner mitochondrial membrane mitophagy receptor. Cell 168, 224–238.e10. 10.1016/j.cell.2016.11.042 28017329 PMC5235968

[B179] Weil Z. M. Corrigan J. D. Karelina K. (2016). Alcohol abuse after traumatic brain injury: experimental and clinical evidence. Neurosci. Biobehav Rev. 62, 89–99. 10.1016/j.neubiorev.2016.01.005 26814960

[B180] Wild P. Farhan H. Mcewan D. G. Wagner S. Rogov V. V. Brady N. R. (2011). Phosphorylation of the autophagy receptor optineurin restricts salmonella growth. Science 333, 228–233. 10.1126/science.1205405 21617041 PMC3714538

[B181] Williams J. A. Ding W. X. (2015a). A mechanistic review of mitophagy and its role in protection against alcoholic liver disease. Biomolecules 5, 2619–2642. 10.3390/biom5042619 26501336 PMC4693250

[B182] Williams J. A. Ding W. X. (2015b). Targeting Pink1-Parkin-mediated mitophagy for treating liver injury. Pharmacol. Res. 102, 264–269. 10.1016/j.phrs.2015.09.020 26655101 PMC4684418

[B183] Williams J. A. Ding W. X. (2018). Mechanisms, pathophysiological roles and methods for analyzing mitophagy - recent insights. Biol. Chem. 399, 147–178. 10.1515/hsz-2017-0228 28976892 PMC5835338

[B184] Williams J. A. Ni H. M. Ding Y. Ding W. X. (2015a). Parkin regulates mitophagy and mitochondrial function to protect against alcohol-induced liver injury and steatosis in mice. Am. J. Physiol. Gastrointest. Liver Physiol. 309, G324–G340. 10.1152/ajpgi.00108.2015 26159696 PMC4556950

[B185] Williams J. A. Ni H. M. Haynes A. Manley S. Li Y. Jaeschke H. (2015b). Chronic deletion and acute knockdown of parkin have differential responses to acetaminophen-induced mitophagy and liver injury in mice. J. Biol. Chem. 290, 10934–10946. 10.1074/jbc.M114.602284 25752611 PMC4409255

[B186] Wong Y. C. Holzbaur E. L. (2014). Optineurin is an autophagy receptor for damaged mitochondria in parkin-mediated mitophagy that is disrupted by an ALS-Linked mutation. Proc. Natl. Acad. Sci. U. S. A. 111, E4439–E4448. 10.1073/pnas.1405752111 25294927 PMC4210283

[B187] Wu C. Chen H. Zhuang R. Zhang H. Wang Y. Hu X. (2021). Betulinic acid inhibits pyroptosis in spinal cord injury by augmenting autophagy *via* the AMPK-mTOR-TFEB signaling pathway. Int. J. Biol. Sci. 17, 1138–1152. 10.7150/ijbs.57825 33867836 PMC8040310

[B188] Wu D. Wang X. Zhou R. Yang L. Cederbaum A. I. (2012). Alcohol steatosis and cytotoxicity: the role of cytochrome P4502E1 and autophagy. Free Radic. Biol. Med. 53, 1346–1357. 10.1016/j.freeradbiomed.2012.07.005 22819980 PMC3436962

[B189] Wu H. Xue D. Chen G. Han Z. Huang L. Zhu C. (2014a). The BCL2L1 and PGAM5 axis defines hypoxia-induced receptor-mediated mitophagy. Autophagy 10, 1712–1725. 10.4161/auto.29568 25126723 PMC4198357

[B190] Wu W. Tian W. Hu Z. Chen G. Huang L. Li W. (2014b). ULK1 translocates to mitochondria and phosphorylates FUNDC1 to regulate mitophagy. EMBO Rep. 15, 566–575. 10.1002/embr.201438501 24671035 PMC4210082

[B191] Xiao Y. Zhou Y. Lu Y. Zhou K. Cai W. (2018). PHB2 interacts with LC3 and SQSTM1 is required for bile acids-induced mitophagy in cholestatic liver. Cell Death Dis. 9, 160. 10.1038/s41419-017-0228-8 29416008 PMC5833850

[B192] Xie X. Li F. Wang Y. Wang Y. Lin Z. Cheng X. (2015). Molecular basis of ubiquitin recognition by the autophagy receptor CALCOCO2. Autophagy 11, 1775–1789. 10.1080/15548627.2015.1082025 26506893 PMC4824588

[B193] Xie Z. Klionsky D. J. (2007). Autophagosome formation: core machinery and adaptations. Nat. Cell Biol. 9, 1102–1109. 10.1038/ncb1007-1102 17909521

[B194] Yamada T. Murata D. Adachi Y. Itoh K. Kameoka S. Igarashi A. (2018). Mitochondrial stasis reveals p62-Mediated ubiquitination in parkin-independent mitophagy and mitigates nonalcoholic fatty liver disease. Cell Metab. 28, 588–604.e5. 10.1016/j.cmet.2018.06.014 30017357 PMC6170673

[B195] Yan C. Gong L. Chen L. Xu M. Abou-Hamdan H. Tang M. (2020). PHB2 (prohibitin 2) promotes PINK1-PRKN/Parkin-dependent mitophagy by the PARL-PGAM5-PINK1 axis. Autophagy 16, 419–434. 10.1080/15548627.2019.1628520 31177901 PMC6999623

[B196] Yao J. Wang J. Xu Y. Guo Q. Sun Y. Liu J. (2022). CDK9 inhibition blocks the initiation of PINK1-PRKN-mediated mitophagy by regulating the SIRT1-FOXO3-BNIP3 axis and enhances the therapeutic effects involving mitochondrial dysfunction in hepatocellular carcinoma. Autophagy 18, 1879–1897. 10.1080/15548627.2021.2007027 34890308 PMC9450969

[B197] Yao Z. Q. Zhang X. Zhen Y. He X. Y. Zhao S. Li X. F. (2018). A novel small-molecule activator of Sirtuin-1 induces autophagic cell death/mitophagy as a potential therapeutic strategy in glioblastoma. Cell Death Dis. 9, 767. 10.1038/s41419-018-0799-z 29991742 PMC6039470

[B198] Ye H. Nelson L. J. Gómez Del Moral M. Martínez-Naves E. Cubero F. J. (2018). Dissecting the molecular pathophysiology of drug-induced liver injury. World J. Gastroenterol. 24, 1373–1385. 10.3748/wjg.v24.i13.1373 29632419 PMC5889818

[B199] Yoo Y. S. Park Y. J. Lee H. S. Oanh N. T. K. Cho M. Y. Heo J. (2019). Mitochondria ubiquitin ligase, MARCH5 resolves hepatitis B virus X protein aggregates in the liver pathogenesis. Cell Death Dis. 10, 938. 10.1038/s41419-019-2175-z 31819032 PMC6901512

[B200] Yoshii S. R. Kishi C. Ishihara N. Mizushima N. (2011). Parkin mediates proteasome-dependent protein degradation and rupture of the outer mitochondrial membrane. J. Biol. Chem. 286, 19630–19640. 10.1074/jbc.M110.209338 21454557 PMC3103342

[B201] Younes R. Bugianesi E. (2019). A spotlight on pathogenesis, interactions and novel therapeutic options in NAFLD. Nat. Rev. Gastroenterol. Hepatol. 16, 80–82. 10.1038/s41575-018-0094-6 30559444

[B202] Yu X. Hao M. Liu Y. Ma X. Lin W. Xu Q. (2019). Liraglutide ameliorates non-alcoholic steatohepatitis by inhibiting NLRP3 inflammasome and pyroptosis activation *via* mitophagy. Eur. J. Pharmacol. 864, 172715. 10.1016/j.ejphar.2019.172715 31593687

[B203] Yu X. Xu Y. Zhang S. Sun J. Liu P. Xiao L. (2016). Quercetin attenuates chronic ethanol-induced hepatic mitochondrial damage through enhanced mitophagy. Nutrients 8, 27. 10.3390/nu8010027 26742072 PMC4728641

[B204] Yu Z. Guo J. Hu M. Gao Y. Huang L. (2020). Icaritin exacerbates mitophagy and synergizes with doxorubicin to induce immunogenic cell death in hepatocellular carcinoma. ACS Nano 14, 4816–4828. 10.1021/acsnano.0c00708 32188241

[B205] Yuan Y. Zheng Y. Zhang X. Chen Y. Wu X. Wu J. (2017). BNIP3L/NIX-mediated mitophagy protects against ischemic brain injury independent of PARK2. Autophagy 13, 1754–1766. 10.1080/15548627.2017.1357792 28820284 PMC5640199

[B206] Zhang R. Chu K. Zhao N. Wu J. Ma L. Zhu C. (2019). Corilagin alleviates nonalcoholic fatty liver disease in high-fat diet-induced C57BL/6 mice by ameliorating oxidative stress and restoring autophagic flux. Front. Pharmacol. 10, 1693. 10.3389/fphar.2019.01693 32116684 PMC7011087

[B207] Zhang Z. Xu T. Chen J. Shao Z. Wang K. Yan Y. (2018). Parkin-mediated mitophagy as a potential therapeutic target for intervertebral disc degeneration. Cell Death Dis. 9, 980. 10.1038/s41419-018-1024-9 30250268 PMC6155159

[B208] Zhao H. Liu S. Zhao H. Liu Y. Xue M. Zhang H. (2021a). Protective effects of fucoidan against ethanol-induced liver injury through maintaining mitochondrial function and mitophagy balance in rats. Food Funct. 12, 3842–3854. 10.1039/d0fo03220d 33977968

[B209] Zhao Y. Zhou L. Li H. Sun T. Wen X. Li X. (2021b). Nuclear-encoded lncRNA MALAT1 epigenetically controls metabolic reprogramming in HCC cells through the mitophagy pathway. Mol. Ther. Nucleic Acids 23, 264–276. 10.1016/j.omtn.2020.09.040 33425485 PMC7773746

[B210] Zheng Y. Huang C. Lu L. Yu K. Zhao J. Chen M. (2021). STOML2 potentiates metastasis of hepatocellular carcinoma by promoting PINK1-mediated mitophagy and regulates sensitivity to lenvatinib. J. Hematol. Oncol. 14, 16. 10.1186/s13045-020-01029-3 33446239 PMC7807703

[B211] Zhou H. Zhu P. Wang J. Toan S. Ren J. (2019a). DNA-PKcs promotes alcohol-related liver disease by activating Drp1-related mitochondrial fission and repressing FUNDC1-required mitophagy. Signal Transduct. Target Ther. 4, 56. 10.1038/s41392-019-0094-1 31839999 PMC6895206

[B212] Zhou T. Chang L. Luo Y. Zhou Y. Zhang J. (2019b). Mst1 inhibition attenuates non-alcoholic fatty liver disease *via* reversing Parkin-related mitophagy. Redox Biol. 21, 101120. 10.1016/j.redox.2019.101120 30708325 PMC6357900

[B213] Zhou Y. Wu R. Wang X. Jiang Y. Xu W. Shao Y. (2022). Activation of UQCRC2-dependent mitophagy by tetramethylpyrazine inhibits MLKL-Mediated hepatocyte necroptosis in alcoholic liver disease. Free Radic. Biol. Med. 179, 301–316. 10.1016/j.freeradbiomed.2021.11.008 34774698

[B214] Zhu Y. Massen S. Terenzio M. Lang V. Chen-Lindner S. Eils R. (2013). Modulation of serines 17 and 24 in the LC3-interacting region of Bnip3 determines pro-survival mitophagy *versus* apoptosis. J. Biol. Chem. 288, 1099–1113. 10.1074/jbc.M112.399345 23209295 PMC3542995

[B215] Zong W. X. Rabinowitz J. D. White E. (2016). Mitochondria and cancer. Mol. Cell 61, 667–676. 10.1016/j.molcel.2016.02.011 26942671 PMC4779192

